# AIM2 promotes renal cell carcinoma progression and sunitinib resistance through FOXO3a-ACSL4 axis-regulated ferroptosis

**DOI:** 10.7150/ijbs.79853

**Published:** 2023-02-13

**Authors:** Qi Wang, Su Gao, Yi Shou, Yujie Jia, Zhihao Wei, Yuenan Liu, Jian Shi, Daojia Miao, Qi Miao, Chuanyi Zhao, Chenchen Liu, Hongmei Yang, Tianbo Xu, Xiaoping Zhang

**Affiliations:** 1 Department of Urology, Union Hospital, Tongji Medical College, Huazhong University of Science and Technology, Wuhan, China.; 2 Institute of Urology, Union Hospital, Tongji Medical College, Huazhong University of Science and Technology, Wuhan, China.; 3 Shenzhen Huazhong University of Science and Technology Research Institute, Shenzhen, China.; 4 Department of Geriatrics, Union Hospital, Tongji Medical College, Huazhong University of Science and Technology, Wuhan, China; 5 Institute of Gerontology, Union Hospital, Tongji Medical College, Huazhong University of Science and Technology, Wuhan, China; 6 Department of Pathogenic Biology, School of Basic Medicine, Tongji Medical College, Huazhong University of Science and Technology, Wuhan, 430030, Hubei Province, China.; 7 Department of Cardiology, Union Hospital, Tongji Medical College, Huazhong University of Science and Technology, Wuhan, China.

**Keywords:** Renal cell carcinoma, Absent in melanoma2, Ferroptosis, Sunitinib resistance, ACSL4

## Abstract

Renal cell carcinoma (RCC) is a serious threat to people's health due to its rapid progression, and patients easily develop resistance to targeted therapy. The absent in melanoma 2 (AIM2) is a receptor protein that has recently been proposed to play an important role in various diseases. In this study, AIM2 was identified as a new biomarker of RCC and promoted RCC progression and sunitinib resistance in an inflammasome-independent manner. Mechanistically, AIM2 promoted FOXO3a phosphorylation and proteasome degradation, thereby reducing its transcriptional effect on ACSL4 and inhibiting ferroptosis. In summary, AIM2 promoted RCC progression and sunitinib resistance through FOXO3a-ACSL4 axis-regulated ferroptosis, which could provide new ideas and therapeutic targets for RCC diagnosis and treatment.

## Introduction

Renal cell carcinoma (RCC) is one of the most common fatal malignant tumors of the urinary system with high morbidity and mortality risks^1^, ^2^. The two most common subtypes are clear cell renal cell carcinoma (ccRCC) and papillary renal cell carcinoma (pRCC), accounting for about 80% and 15%, respectively^3^. Due to insensitivity to radiotherapy and chemotherapy and concealment of early clinical manifestations, many patients are diagnosed with advanced or metastatic RCC^4^. Recently, although treatment with molecular targeted drugs, such as sunitinib, have improved the prognosis of some patients, most patients will eventually develop drug resistance^5^. Therefore, an in-depth study of the molecular mechanism of the occurrence and development of RCC is necessary and helpful to find new therapeutic targets.

An inflammatory body (inflammation) is a kind of protein complex, which is composed of receptor protein, junction protein, and aspartate protease-1 (procaspase-1)^6^. The abnormal expression and activation of inflammatory bodies play an important role in the induction and progression of tumors^6^. Recently, there have been many studies on AIM2 inflammatory bodies, a member of the ALR family, which is mainly composed of HIN-200 and PYD domains. It has been reported that the abnormal activation of AIM2 inflammatory bodies could inhibit tumor immunity and was related to the occurrence of glioblastoma and lung cancer^7^, ^8^. However, AIM2 inflammatory bodies are rarely reported in RCC.

Ferroptosis is a novel type of cell death characterized by peroxidation of polyunsaturated fatty acids (PUFAs) and changes in mitochondrial morphology and function^9^. Ferroptosis is associated with the occurrence and development of many tumors^10^. Recent studies have shown that ferroptosis is involved in the chemoresistance mechanism of tumors^11^, ^12^. However, the regulatory mechanism of ferroptosis in RCC has not been fully studied, and the relationship between ferroptosis and tyrosine kinase inhibitor (TKI) therapy resistance has not been reported.

Our study identified AIM2 as a new biomarker of RCC. AIM2 was overexpressed and found to promote RCC progression and sunitinib resistance. Mechanistically, AIM2 promotes FOXO3a phosphorylation and proteasome degradation, thereby reducing its transcriptional effect on ACSL4 and inhibiting ferroptosis.

## Materials and methods

### Data download and bioinformatics analysis

Transcriptomic and clinical information of 530 ccRCC patients in TCGA-KIRC project were downloaded from UCSC Xena. We analyzed relevant data with R 4.0.2, GraphPad Prism 8.4.2, and SPSS 25.0.

The differentially expressed genes (DEGs) was identified using “limma” package with p-value < 0.05 and |log2FC| > 3. Then, DEGs were used for Weighted Gene Co-expression Network analysis (WGCNA). geneModuleMembership (Absolute value > 0.8) and geneTraitSignificance (Absolute value > 0.2) were used to identify hub genes 13, 14.

We used Gene Set Enrichment Analysis (GSEA) to determine interested enrichment pathways with GSEA software (v4.1.0, USA) based on TCGA-KIRC project. The screening criteria was FDR < 0.25 and P < 0.05.

### Human RCC tissues and cell lines

The American Type Culture Collection (ATCC, USA) provided the RCC cell lines (786O, OSRC2, Caki-1, A498 and ACHN) and the normal renal tubular epithelial cell line HK-2. We added 10% fetal bovine serum (FBS) to DMEM ((Gibco, MA, USA)) to make cell culture medium. According to our previous research^13^, 120 pairs of RCC and corresponding normal renal tissues collected from KIRC patients in Wuhan were involved in our study and their clinical information was summarized in the table S1. The Human Research Ethics Committee of Huazhong University of Science and Technology have approved our research. Our research complied with Declaration of Helsinki.

### Immunohistochemistry and immunofluorescence staining

Tissues were immersed in 4% paraformaldehyde (PFA) for fixation. Dehydration was performed using 75%, 85%, 90% and 100% ethanol, respectively. The tissues were later transparent with xylene. The tissues were then fixed in paraffin wax and cut into 4 um-thick slices using a microtome. Before being closed with 3% hydrogen peroxide, the tissue slices were deparaffinized, rehydrated, and antigen-repaired. The tissue sections were incubated with the corresponding primary antibodies for 8 hours. Immune complexes and cell nuclei were visualized with DAB and hematoxylin (BS915, Biosharp, China), respectively. Pictures were obtained using ordinary light microscope (Jiangnan, China).

Immunohistochemistry (IHC) scores are divided into two aspects, staining intensity and percentage of positive cells. With ratings of 0, 1, 2, and 3, the four staining intensity levels are none, weak, moderate, and strong. The percentage of positive cells also has four levels: 0-25%, 25-50%, 50-75%, and 75-100%, with scores of 1,2,3,4, respectively. IHC score = score of staining intensity * score of percentage of positive cells.

For immunofluorescence staining, round coverslips (WG654, Servicebio, China) were placed in the wells of 24-well plates, and then cells were passaged into the 24-well plates. When cell fusion reached about 10%, we fixed cells using 4% PFA. After that, the cells were subjected to membrane breakage and antigen closure. The appropriate mixed primary antibody was applied to the cells and incubated there for 8 hours at 4°C. Then, fluorescent secondary antibodies (B40941 and A-21428, Thermo, USA) were used. DAPI (D9542, SIGMA, USA) was used to label cell nuclei. With the use of a laser confocal microscope (Zeiss, Thornwood, NY), pictures were taken. See table S2 for information about primary antibodies.

### RT-PCR and Western Blotting

Following the manufacturer's instructions, we used the Ultrapure RNA kit (CW0581M, CWBIO, China) to extract tissue and cellular total RNA. 1ug of extracted RNA was utilized for reverse transcription using ABScript III RT Mix (RK20429, Abclonal, China) after measuring the concentration. Real-time qPCR was performed utilizing the produced cDNA, SYBR Green Mix (A25779, Thermo, USA) and StepOnePlus™ PCR system (Bio-rad, USA). To make the expression of the target genes normal, GAPDH was utilized as an internal reference. See table S2 for information about primer sequences. The target gene expression levels for three experiments were expressed using the mean ± standard deviation (SD).

For western blotting, we used RIPA lysis buffer (P0013C, Beyotime, China) supplemented with phenylmethanesulfonylfluoride (PMSF, #ST506, Beyotime, China) and phosphorylated protein inhibitors (G2007, Servicebio, China) to lyse tissues and cells. Ultrasound was used to disrupt the structure of the cell nucleus during the approximately 30 min lysis. The lysates were centrifuged, and the BCA Protein Assay Kit (P0010S, Beyotime, China) was used to determine the amount of protein in the supernatant. Electrophoresis was performed with 15-20 ug of protein in 10% polyacrylamide gel (PG112, Yamei, China) and then transferred to polyvinylidene difluoride membranes (PVDF). After blocked with 5% milk buffer for 90 mins, membrane were treated with corresponding primary antibodies for 8 h at 4°C and corresponding secondary antibodies (AS014 and AS003, Abclonal, China) for 1.5 h at 25°C. Finally, the membranes were exposed with a gel imaging system (Bio-Rad, USA). Information about the primary antibodies is shown in table S2.

### CCK-8 assays, cell viability assays and Edu assays

For the CCK-8 assay, cells were trypsinized, centrifuged and counted, and then passaged into 96-well plates at 2 × 10^3^ cells per well with four replicates per group. Using the CCK-8 kit (HY-K0301, MCE, USA), cell viability was assessed at 450 nm for all groups at 24, 48, 72, and 96 h in accordance with the manufacturer's instructions.

Cells were passaged into 96-well plates at a density of 2*10^3^ cells per well with four replicates per group to test for cell viability. 24 h later, drug treatment was performed. Then, after another 48 hours, cell activity was measured at 450 nm with the CCK8 kit.

For Edu assays, the method was similar to previous studies^15^. When 50% confluency was reached, cells were fixed, and then dyed with the Edu kit (C0075S, Beyotime, China) in accordance with the manufacturer's protocol. Following air-drying, photographs were taken with a 200x fluorescence microscope (KEYENCE, Japan). The red color cells represented proliferating cells. The ability of the cells to proliferate was evaluated using the red cell percentage. Each group was repeated 3 times for statistical purposes.

### Transwell assays

Cells were starved for 12 hours in serum-free medium, then trypsinized, centrifuged, and counted. Resuspend cells in serum-free DMEM. Add 600ul of cell culture medium to each well of a 24-well plate, put transwell®inserts (01020023, Corning, USA) into the wells, and then add 2*104 ACHN or 4*104 Caki-1 cells for migration experiments. For invasion, 60ul of diluted matrigel (354234, Corning, USA) was previously added to the upper layer of the insert before adding double the number of cells. After 24 hours, the cells on the lower surface of the insert were fixed with 4% PFA for 10 minutes, then stained with methyl violet for 30 minutes. The cells on the upper surface were taken out with a cotton swab. Three images per group were randomly selected for cell counting.

### BODIPY C11 assays and flow cytometry analysis

Cells were cultured in 96-well plates. When cell fusion reached approximately 50%, cells were treated with the C11 bodipy working solution for 30 minutes at 37°C. The working solution was serum-free DMEM containing 2uM C11 bodipy (D3861, Thermo, USA). Then, cells were stained with the DAPI (D9542, Sigma, USA). Then, cells were used to take pictures with a fluorescence microscope at 200X.

Flow cytometry analysis: cells were cultivated in six-well plates. When cell fusion reached approximately 80%, C11 bodipy working solution was used to treat cells for 30 minutes at 37°C. Then, cells were trypsinized, centrifuged, and resuspended in 300ul PBS per well. The levels of Lipid ROS were examined using a flow cytometer on FITC channel. The results are processed using FlowJo V10.5.2 software (BD, USA). Three replicate samples per group were used for statistics.

### Transmission electron microscopy

Cells were cultured in 10 cm culture dishes. The culture medium was poured directly from the cell surface and a sufficient amount of pre-cooled 2.5% glutaraldehyde was immediately added for rapid fixation for 1 hour. The cells were then quickly scraped off with a cell spatula and all transferred to a centrifuge tube and centrifuged at 650 rpm. Next, the supernatant was eliminated. The cells were then fixed with 1% OsO4 after being rinsed with 0.1 M phosphate buffer. Cell pellets were dehydrated and embedded with resin to make 60-80 nm sections of the cells. Cells were examined using an HT7800 transmission electron microscope (JEOL, Tokyo, Japan) after being stained with uranyl acetate and lead nitrate. In each condition, five areas were randomly selected for high-resolution digital images.

### Lentivirus construction and infection

Genechem Co. Ltd (China) offered AIM2 overexpression and knockdown lentivirus and FOXO3a knockdown lentivirus. The vector of lentivirus was plvs-ires-puro. Cells were infected with corresponding lentivirus following the manufacturer's instructions. Specific sequences of shRNA were:

shAIM2-1: GGAACAATTGTGAATGGTTTG;

shAIM2-2: CCCGAAGATCAACACGCTTCA;

shFOXO3a-1: GCACAACCTGTCACTGCATAG;

shFOXO3a-2: GGAACTGGCAAGAGCTCTTGG

### Tumor formation assay

ACHN and Caki-1 cells infected with the corresponding lentivirus were used for tumor formation assay. Cells were trypsinized, centrifuged, counted, and resuspended in PBS, maintaining a cell density of 2.5*107 cells per 1 ml of PBS. Then, each 5-week-old nude mouse was injected subcutaneously with 200ul of cell suspension. Charles River Laboratories (Beijing, China) provided the mice. Measure tumor growth every five days with a digital caliper. The long diameter is represented by D and the short diameter is represented by d. Tumor volume = (D * d2)/2. The mice were euthanized after 25-30 days and the tumor weight was then calculated.

### *In vivo* carcinoma metastasis assay

After infected with the corresponding lentivirus, ACHN and Caki-1 cells were used for the caudal vein metastasis model. Similar to tumor formation assay, 2.5 × 10^6^ cells suspended in 100ul PBS were injected into the tail vein of mice. Approximately 7 weeks later, all mice were anesthetized and small animal live imaging was conducted with the Spectral Instruments Imaging System (Tucson, USA). The images were processed by Bruker MI SE 721 software (Bruker, Germany). Then, all mice were euthanized. The liver tissues were photographed and fixed with 4% PFA for H&E staining.

### MDA assay

Fresh subcutaneous tumor tissues were used to measure lipid oxidation levels using the MDA kit (S0131S, Beyotime, China). Briefly, directed by the manufacturer's instructions. Various standard concentrations were used to build a standard curve. The tissues were lysed using lysis solution buffer. The lysates were reacted with the MDA assay working solution. At 532nm, the absorbance was finally measured. Lipid peroxidation levels in tumor tissues were determined according to a standard curve and normalized using protein concentrations as a control. The results of three independent experiments were collected for statistical analysis.

### Construction of sunitinib-resistant RCC cell lines

We used a drug concentration increment method to construct ACHN and Caki-1 resistant cell lines. Briefly, we used 5uM sunitinib as the starting concentration. During the logarithmic growth of the cells, we added 5uM sunitinib to the medium for 24 hours and then switched to normal medium. After the cells recovered their status, we added 5uM sunitinib again. We repeated the above dosing experiments until 5uM sunitinib was fully tolerated by the cells. Then the drug concentration was increased to 7um and a new round of drug tolerance assay was performed. Finally, the IC50 of drug-resistant cells was examined and used to determine whether the drug-resistant cells were successfully constructed.

### Chromatin immunoprecipitation (ChIP) assays

We performed ChIP assays followed by previous study^16^. According to the protocol provided by manufactures, ChIP Kit (#9002, CST, USA) was used to perform CHIP assays. Overall, the ChIP assays consisted of three steps: cell culture cross-linking and sample preparation, cell nuclei preparation and chromatin digestion, and chromatin immunoprecipitation. The primer sequences information was below.

E1 and E2: 3′-TCCAAGTAGTTTCTTGGTCTGAT -5′, 3′-TCACTATAGCCTGAAACTATCCGT-5′; E3: 3′-ACCCAGTGAATTACGTACCTG-5′, 3′-AGACAGTCATGTAGCTTCCTTT -5′.

Polyclonal FOXO3a antibody and IgG antibody were used for ChIP. Primary DNA was used as a positive control.

### Dual luciferase reporter assays

GV550 was used for plasmid vector. E1 (ATAAACAT) and E2 (TTGTATAC) were mutated to MUT1 (GCGGGTGC) and MUT2 (CCACGCGT), respectively. 293T cells were co-transfected with five truncated or mutant plasmids and shFOXO3a-1 plasmid. Promega (Madison, USA) provided a dual luciferase reporter assay kit (E1910, Promega, China), which was used to detect luciferase activity. We used renilla luciferase as a control to normalize luciferase activity. Results from three independent experiments were collected for statistical analysis.

### Cycloheximide (CHX) chase assays

Cells were treated with 100 μM cyclohexanone (CHX) for 1, 2, 3, 4 and 5 h, respectively. Then, cells were collected for protein extraction and western blots. Levels of protein were quantified using image lab software (Bio-rad, USA).

### MG132 and chloroquine treatment

MG132 was an inhibitor of the proteasomal pathway of protein degradation, while chloroquine was an inhibitor of the lysosomal pathway. MG132 and chloroquine were used to treat ACHN and Caki-1 cells for 24h, respectively. Then, the cells were collected for protein extraction and western blots.

### Statistical analysis

Group data were presented as mean and standard deviation (SD). The data were tested for normality before the subsequent analysis. Differences between groups were determined using a t-test. The correlation between AIM2 and clinicopathological traits was examined using Pearson's χ2 test. Univariate and multivariate Cox regression analyses were used to determine AIM2 as an independent risk factor for patients with RCC. p < 0.05 was significant. All *in vitro* experiments were repeated 3 times. GraphPad Prism 8.4.2 (GraphPad, USA) and SPSS 25.0 (SPSS, USA) were used for the above analyses.

## Results

### AIM2 was identified as a biomarker of RCC

**Figure 1:**
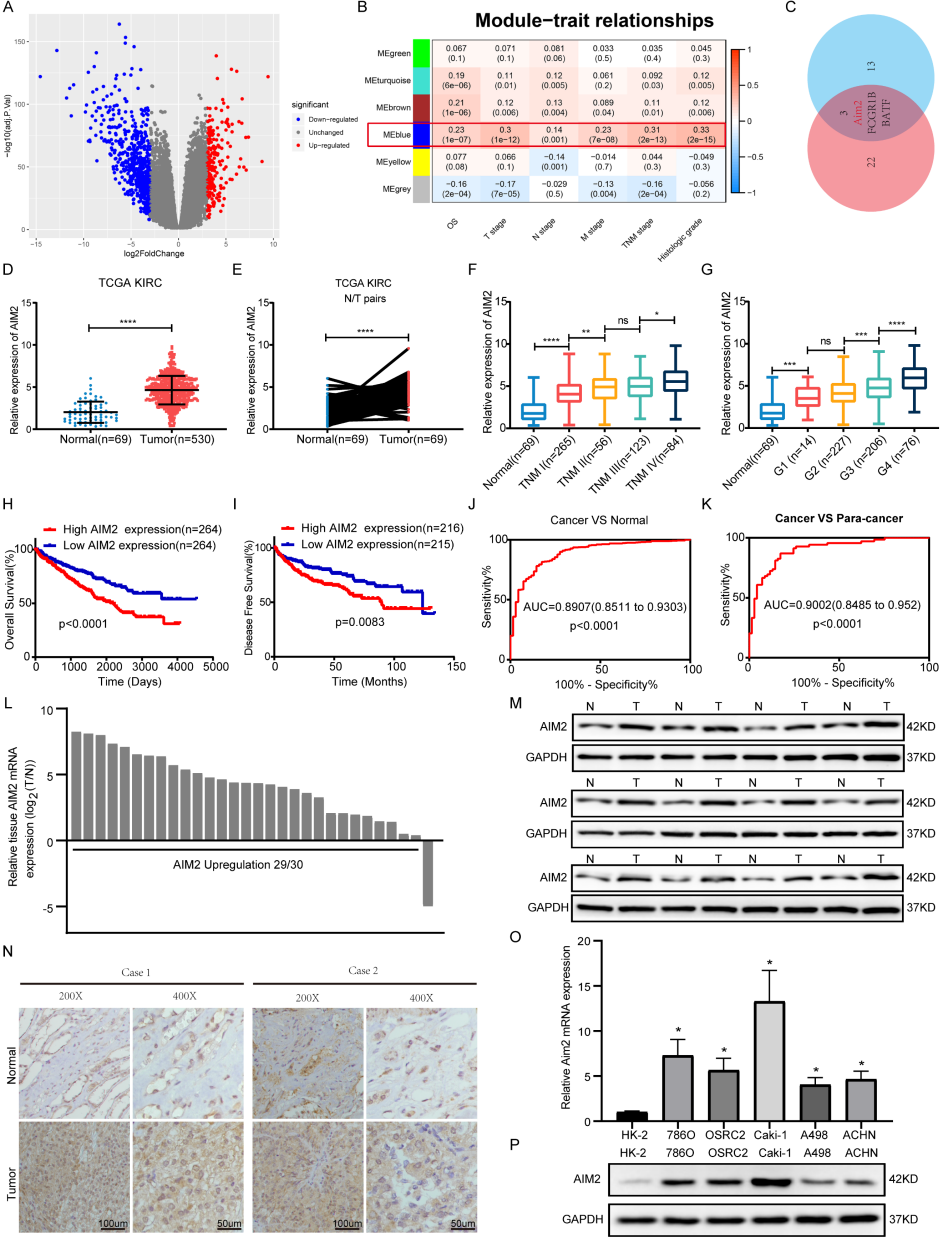
** AIM2 was identified as a biomarker of RCC.** (A) Volcano plots were used to display DEGs, red denoted highly expressed genes with logFC >3 and blue denoted low expressed genes with logFC < -3. (B) clinical features including OS, T, N, M, TNM stage, and histologic grade are related to six modules. The blue module was the most important one. (C) Venn diagram identified three genes of interest. (D-E) Expression of AIM2 in unpaired and paired tissues from TCGA-KIRC project. (F-G) Expression of AIM2 according to TNM stage and G stage in TCGA-KIRC project. (H-I) The OS and DFS of patients in AIM2 high group and AIM2 low group were visualized by Kaplan-Meier plot. (J-K) The ROC curves for AIM2 in cancer vs normal and cancer vs para-cancer. AIM2 overexpression in RCC was validated through (L) qRT-PCR assays (30 pairs), (M) immunoblotting tests (12 pairs), and (N) immunohistochemical analyses (2 pairs). (O) mRNA expression of AIM2 in RCC and normal cell lines using qRT-PCR. (P) Immunoblotting tests displayed AIM2 expression in RCC cell lines (786-O, A498, Caki-1, ACHN and OSRC-2) compared to normal renal cell line (HK-2). Relative *p < 0.05; **p < 0.01; ***p < 0.001; ****p < 0.0001.

According to the criterion of |logFC| ≥ 3, 771 differentially expressed genes were identified and used for WGCNA in TCGA-KIRC project (Figures 1A and S1A). As shown in Figure 1B, the blue module was mostly related to the clinical traits, including TNM stage, G grade, and overall survival. Two key subsets were identified from the blue module: one subset was screened based on the correlation between genes and modules >0.8, and the other subset was based on the correlation between genes and clinical traits (N stage was not involved) >0.2 (Table S3). By screening the two gene subsets, three genes were thought to be hub genes of the blue module (Figure 1C and S1B). Then, Kaplan-Meier survival analysis and log-rank tests were conducted, and AIM2 was found to be most associated with the prognosis in KIRC patients (Figures S1C-S1E). Transcriptomically, AIM2 was overexpressed in tumor tissues in the TCGA-KIRC project and oncomine database (Figures 1D-1E and S1F-S1J). As shown in figures 1F-1G and S1K-S1M, many clinical stages (T, N, M and TNM, and G stage)were positively correlated with AIM2 expression. Survival analysis showed a better prognosis in AIM2 low expression group (Figures 1H and 1I). The receiver operating characteristic curve (ROC) analysis showed that AIM2 was a good diagnostic marker (Figures 1J and 1K). The AIM2 expression was correlated with survival time tumor grade and stage (Table S4). Univariate and multivariate analyses revealed that AIM2 was an independent prognostic marker of RCC (Table S5). Thus, AIM2 could be used as a potential diagnostic and prognostic biomarker for RCC. To investigate why AIM2 was highly expressed in RCC, we examined its CNV in the TCGA-KIRC project. Altogether, 11.93% of patients had copy number amplification of AIM2, and its CNV was proportional to its expression (Figures S2A and S2B). Furthermore, patients with AIM2 copy number amplification had a worse prognosis than those with unchanged or deleted copy number (Figures S2C-S2E). Therefore, we believe that the overexpression of AIM2 in RCC is related to its copy number amplification.

To verify the high expression of AIM2 in RCC, expression of AIM2 in RCC tissues and cell lines was examined using qRT-PCR and immunoblotting test (IBT) and using CCLE database (Figures 1L, 1M, 1O, 1P and S1N). The immunohistochemistry (IHC) results of the tumors and normal tissues also indicated that AIM2 was increased in cancer tissues (Figure 1N).

### AIM2 promoted the progression of RCC *in vitro*

**Figure 2:**
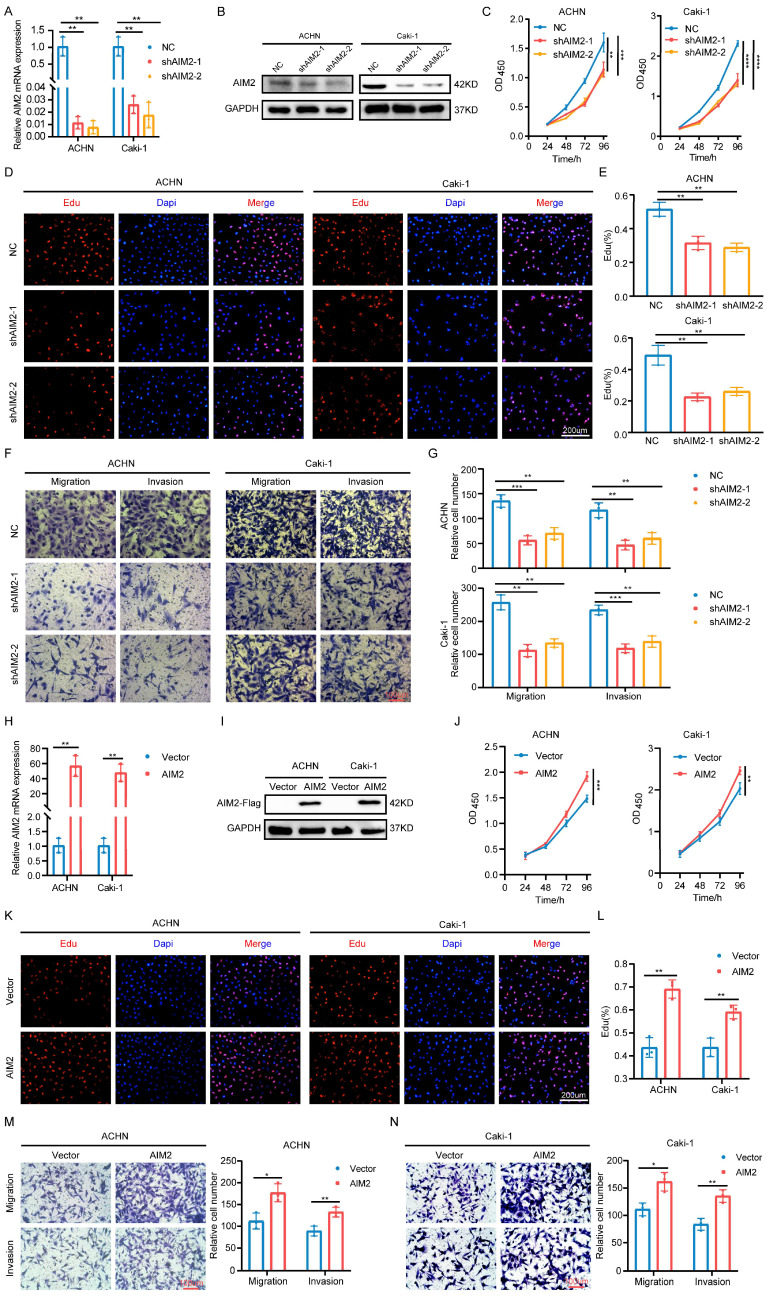
** AIM2 promoted RCC progression *in vitro*.** (A and H) Validation of mRNA levels after introduction of corresponding lentivirus (n = 3). (B, I) Validation of protein levels after introduction of corresponding lentivirus. (C and J) The growth curves of ACHN and Caki-1 cells after knockdown or overexpression of AIM2 were conducted using CCK-8 assays (n = 4). (D, E, K and L) The proliferation ability of ACHN and Caki-1 cells after depleting or overexpressing AIM2 was evaluated using EdU assays (n = 3). (F, G, M and N) The metastasis ability of ACHN and Caki-1 cells after depleting or overexpressing AIM2 was determined by transwell assays (n = 3). Relative *p < 0.05; **p < 0.01; ***p < 0.001; ****p < 0.0001.

To explore the role of AIM2 in RCC, two shRNAs targeting AIM2 were constructed with lentiviral vectors (Figures 2A and 2B). The CCK-8 and EdU assays showed that the proliferation capacity of RCC cells was weakened after AIM2 knockdown (Figures 2C-2E). Additionally, RCC cells' capacity to migrate and invade was hampered by AIM2 knockdown (Figures 2F and 2G). To further verify the functionality of AIM2 in RCC, we successfully constructed ACHN and Caki-1 cell lines with AIM2 stably overexpressed using lentivirus (Figures 2H and 2I). On the contrary, overexpression of AIM2 increased the proliferation and metastatic ability of RCC cells (Figures 2J-2N). These results suggested that AIM2 promoted RCC progression *in vitro*.

### AIM2 promoted the progression of RCC *in vivo*

**Figure 3:**
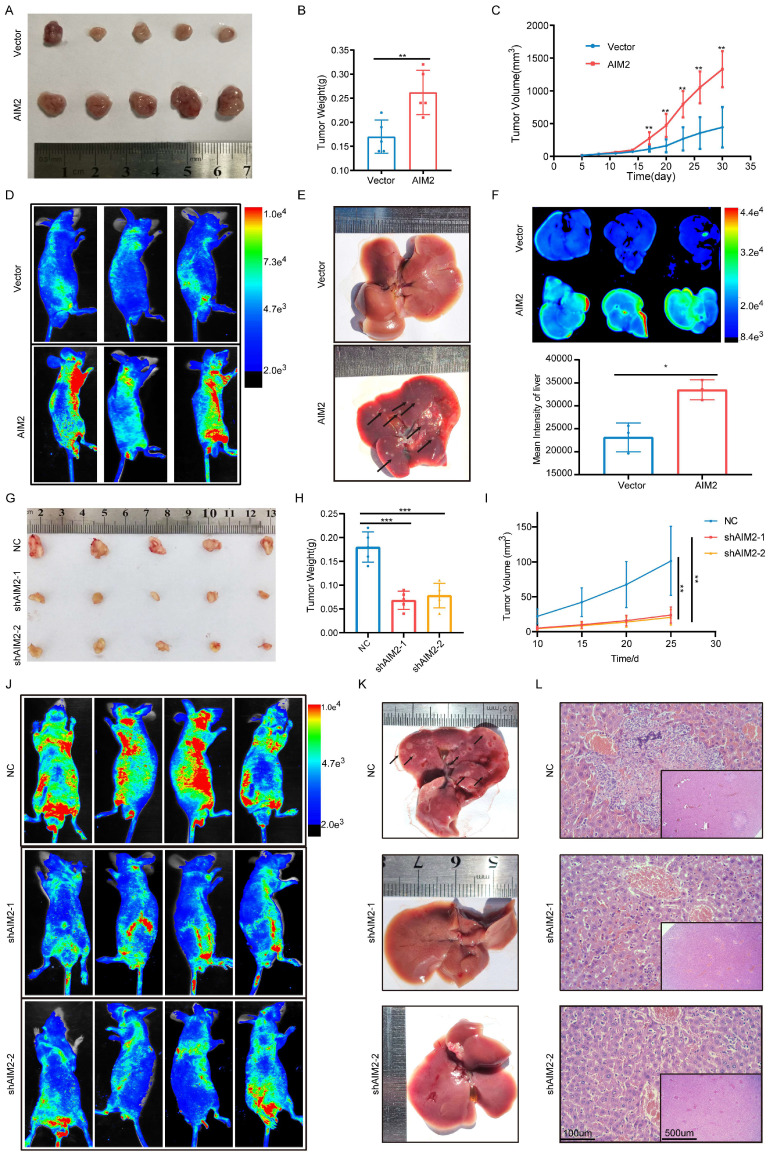
** AIM2 promoted RCC progression *in vivo*.** (A) Pictures of xenografts formed from ACHN cells with or without AIM2 overexpressed (n = 5). (B) Tumor weight of xenografts in the two groups (n = 5). (C) The growth curves of xenograft tumor in the two groups (n = 5). (D) Living fluorescence image in the metastasis mode with or without AIM2 overexpressed. (E-F) The picture and living fluorescence image of livers in the two groups. (G) Pictures of xenografts formed from Caki-1 cells with or without AIM2 knockdown (n = 5). (H-I) Tumor weight and growth curves of xenografts in the three groups (n = 5). (J) Living fluorescence image in the metastasis mode with or without AIM2 knockdown. (K-L) The picture and living fluorescence image of liver in the three groups *p < 0.05; **p < 0.01; ***p < 0.001.

Based on the important role of AIM2 in promoting RCC progression *in vitro*, we further evaluated its role *in vivo*. Considering the lower protein level of AIM2 in ACHN among RCC cell lines (Figure 1P), a xenograft tumor model using AIM2 overexpressing ACHN cells in the axilla of nude mice was established. As shown in Figures 3A-3C, the volume and weight of the tumors with AIM2 overexpression were significantly greater than those in the control group. Additionally, we constructed mice tumor metastasis models by injecting ACHN cells into the tail vein of nude mice.

Small animal live imaging indicated that AIM2 overexpression considerably promoted the level of tumor metastasis (Figures 3D and S3A). Meanwhile, the AIM2 overexpression group was thinner than the control group, which may be caused by excessive tumor metastasis (Figures S3B and S3C). Furthermore, organ live imaging showed that it was in the liver rather than the lung, kidney, or spleen that tumor metastasis was significantly different (Figures 2E-2F and S3D-S3L). Hematoxylin-eosin (HE) and vimentin staining of organ tissue section also supported it (Figures S3M-S3Q). Meanwhile, As the protein level of AIM2 was highest in Caki-1 among RCC cell lines (Figure 1P), the xenograft tumor model and mice tumor metastasis model were constructed using Caki-1 cells with AIM2 knockdown. As expected, AIM2 knockdown significantly inhibited RCC growth and metastasis (Figures 3G-3L). These results suggested that AIM2 promoted RCC proliferation and metastasis *in vivo*.

### AIM2 inhibited RCC ferroptosis

**Figure 4:**
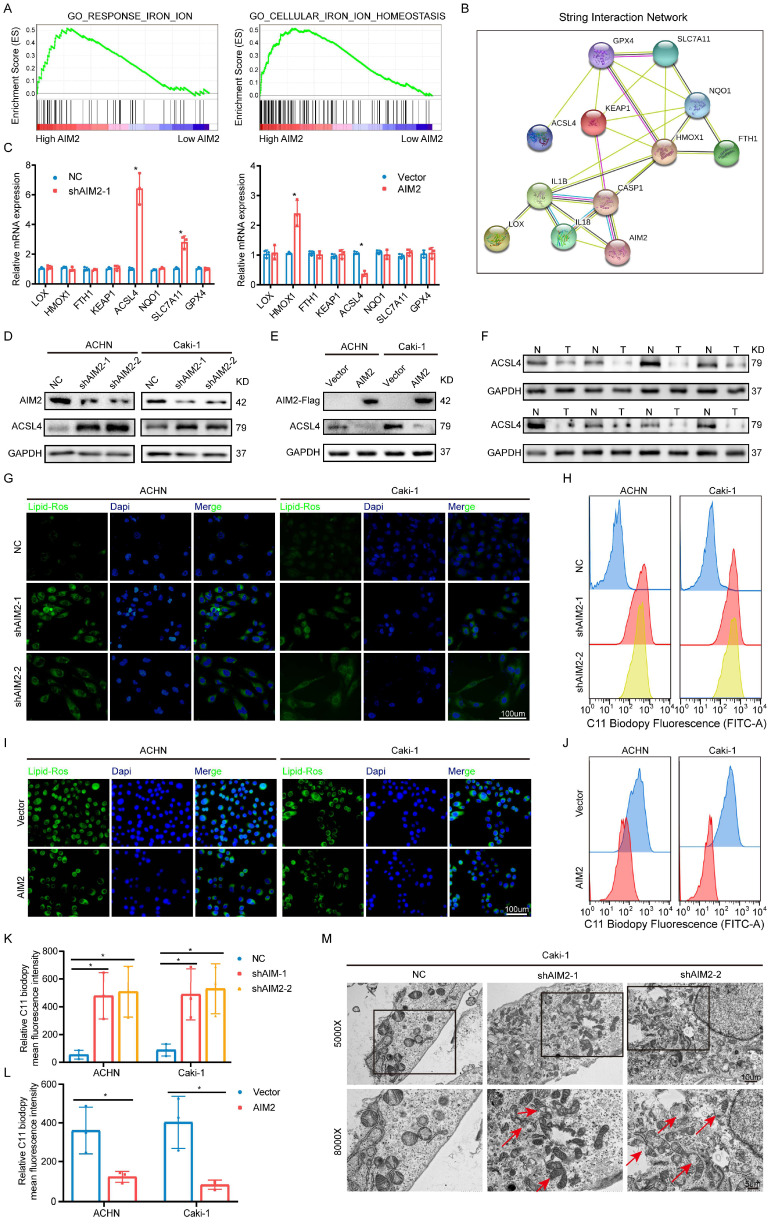
** AIM2 inhibited ferroptosis in RCC.** (A) The GSEA results between AIM2 high and AIM2 low groups using TCGA-KIRC project at the criteria of FDR less than 0.25, P less than 0.05, |NES| > 1. (B) The PPI network of AIM2 using string database. (C) The mRNA levels of the indicated molecules in AIM2 knocked down or overexpressed ACHN cells were determined by qPCR. (D-E) The protein level of ACSL4 was detected by IBT tests in AIM2 knocked down or overexpressed ACHN and Caki-1 cells. (F) The protein level of ACSL4 was detected in eight pairs of RCC and corresponding normal tissues. (G and I) The lipid peroxidation level in ACHN and Caki-1 cells with AIM2 knocked down or overexpressed. (H, J, K and L) The lipid peroxidation levels in ACHN and Caki-1 cells with AIM2 knocked down or overexpressed were detected by flow cytometry using C11-BODIPY 581/591 probe (n = 3). (M) Electron micrographs in Caki-1 cells with AIM2 knocked down. Red arrows indicated mitochondria with membrane damaged, membrane density increased or small size. *p < .05.

Given that AIM2 could function in tumors in an inflammasome-dependent or -independent manner^17^, ^18^, we accessed the status of inflammasome activation after expression of AIM2 was altered. Similar protein expression of caspase-1 and cleaved caspase-1 were detected in ACHN and Caki-1 cells with AIM2 knockdown and overexpression (Figures S4A and S4B), which indicated that AIM2 promoted RCC progression in an inflammasome-independent manner. To investigate the way that AIM2 affected carcinogenesis, GSEA was conducted based on the TCGA-KIRC project. The results showed that AIM2 was involved in iron ion homeostasis and response, which were important parts of ferroptosis (Figure 4A). Then, a protein-protein interaction network indicated that AIM2 was strongly connected to ferroptosis-related genes (Figure 4B). To identify the downstream genes, we detected the mRNA expression levels of these genes after knockdown or overexpression of AIM2 in Caki-1 cells (Figure 4C). ACSL4 was thought to be the only gene negatively regulated by AIM2, and the IBT results also support it (Figures 4D and 4E). We found that ACSL4 was down-regulated in RCC through the TCGA-KIRC project and our own collection of tumor tissues (Figures 4F and S4C). ASCL4 reportedly promotes the formation of phospholipids containing PUFAs, thereby causing ferroptosis^19^. Therefore, as expected, the lipid peroxidation level of cells was increased or decreased after knockdown or overexpression of AIM2 (Figures 4G and 4I). The flow cytometry results also revealed this (Figures 4H, 4J, 4K and 4L). Then, RSL3, a ferroptosis inducer targeting GPX4, was used in our research. Knockdown or overexpression of AIM2 could increase or decrease RSL3 sensitivity in ACHN and Caki-1 cells, respectively (Figures S4D-S4G). Furthermore, the AIM2 knockdown Caki-1 cells showed shrinking mitochondria with increased membrane density, which was a ferroptosis-specific morphology (Figure 4M). Meanwhile, the difference of cell viability caused by AIM2 was abolished when ferrostatin-1, a ferroptosis inhibitor, were applied in ACHN and Caki-1 cells (Figure S4H and S4I). Above all, we believed that AIM2 inhibited ferroptosis in RCC.

### AIM2 inhibited ACSL4 activity through FOXO3a

**Figure 5:**
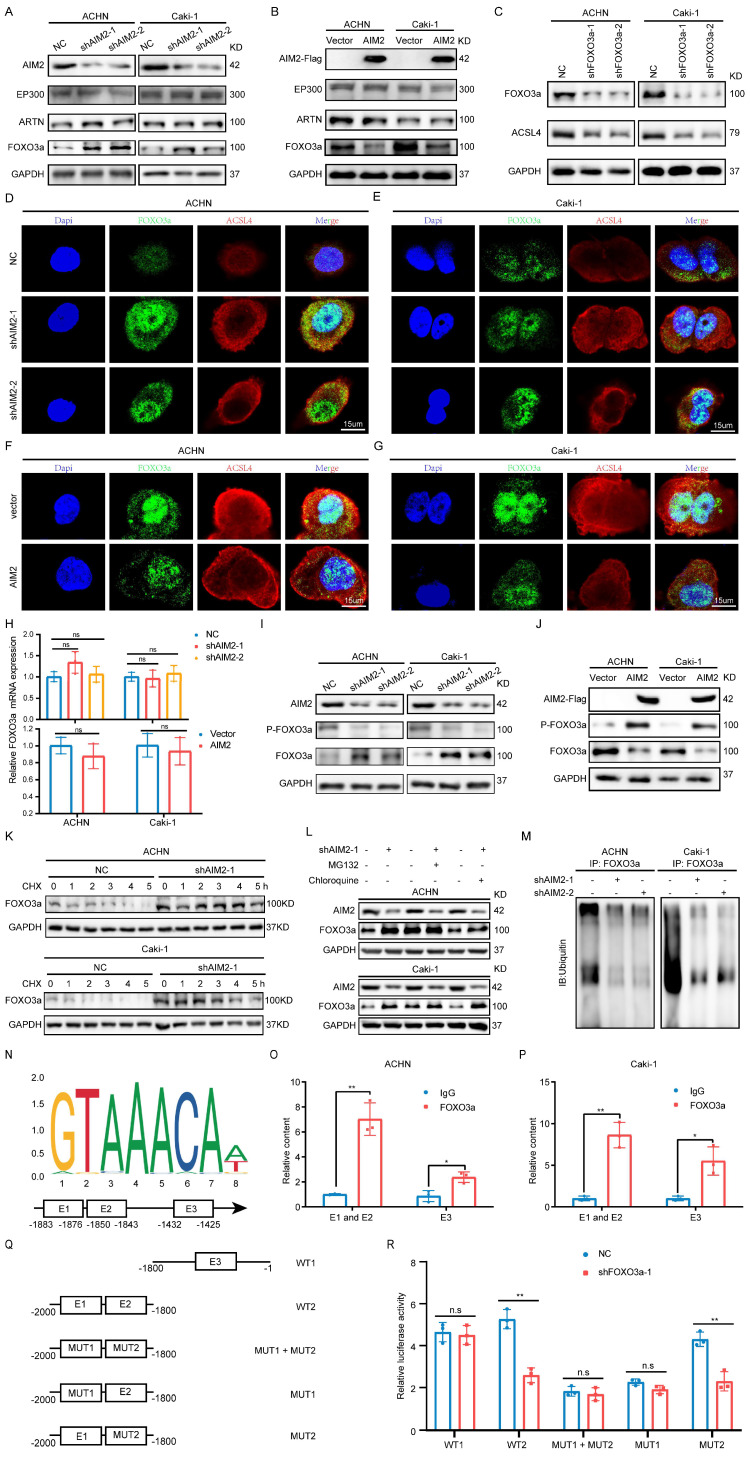
** AIM2 inhibited ACSL4 activity through FOXO3a.** (A-B) The protein levels of EP300, ARTN and FOXO3a in AIM2 knocked down or overexpressed ACHN and Caki-1 cells. (C) The expression of ACSL4 was detected by IBT tests in FOXO3a knocked down RCC cells. (D-G) Representative photographs of immunofluorescence staining for FOXO3a and ACSL4 in the ACHN and Caki-1 cells with AIM2 knockdown and overexpression. (H) The mRNA level of FOXO3a was detected by qPCR in AIM2 knocked down or overexpressed ACHN and Caki-1 cells (n = 3). (I-J) The phosphorylation level of FOXO3a after overexpression or knockdown of AIM2. (K) Protein stability experiments of ACHN and Caki-1 cells with AIM2 knockdown after treated with 100mM cycloheximide (CHX) for 0h, 1h, 2h, 3h, 4h, and 5h. (L) After treated with MG132 (25nM) or chloroquine (70nM) for 24h, RCC cells with AIM2 knockdown were used for IBT. (M) Immunoprecipitation (IP) with anti-FOXO3a antibody was applied in AIM2 knockdown cells, and visualized using western blot assays. (N) The predicted binding sites of FOXO3a to the promoter of ACSL4. (O-P) In ACHN and Caki-1 cells, direct FOXO3a binding to ACSL4 promoter regions was demonstrated by ChIP-PCR experiments (n = 3). (Q) Schematic diagram of five truncated and mutant plasmids. (R) After co-transfected the five truncated or mutant plasmids with shFOXO3a-1 plasmid, 293T cells were used for dual luciferase reporter assays (n = 3). **p < .01 and *p < .05.

Since AIM2 can regulate the mRNA and protein levels of ACSL4, we naturally thought that this occurred through transcriptional regulation. The animal TFDB database was utilized to predict the potential top five transcription factors of ACSL4 (Figure S5A). A correlation analysis with ACSL4 indicated that EP300, ARTN, and FOXO3a were more likely to be transcription factors of ACSL4 (Figures S5B-S5E). We then examined the protein levels of these three transcription factors in AIM2 overexpressing and knockdown cells, and found that FOXO3a seemed to be the transcription factor downstream of AIM2 that promotes the transcription of ACSL4 (Figures 5A and 5B). Subsequently, we found that the expression level of ACSL4 was suppressed after knockdown of FOXO3a in ACHN and Caki-1 cells (Figures 5C and S5F-S5G). The results of immunofluorescence showed that the knockdown of AIM2 resulted in increased expressions of FOXO3a and ACSL4 in the nucleus and cytoplasm, respectively (Figures 5D-5E). Consistently, we observed the decreased expression of FOXO3a and ACSL4 in AIM2-overexpressing cells (Figures 5F and 5G). Therefore, FOXO3a was regulated by AIM2 and appeared to be a transcription factor of ACSL4. To our surprise, the results of qPCR indicated that AIM2 didn't regulate the mRNA level of FOXO3a (Figure 5H), which brought us to post-translational modification of FOXO3a. Phosphorylation and ubiquitination were the two most common post-translational modifications of proteins in cells^20^. Previous studies have shown that phosphorylated FOXO3a was confined to the cytoplasm and modified by ubiquitin for degradation^21^, ^22^.

Therefore, we examined the phosphorylation level of FOXO3a after overexpression or knockdown of AIM2, and the IBT results indicated that AIM2 promoted the phosphorylation of FOXO3a (Figures 5I-5J). After treatment with CHX, the protein level of FOXO3 in the control cells decreased, and this was weakened in AIM2 knockdown cells, suggesting that AIM2 promoted the degradation of FOXO3a (Figures 5K and S5H-S5I). To determine whether the proteasome pathway or the lysosomal pathway mediated FOXO3a degradation^23^, MG132 and chloroquine were used to identify the specific mechanism of regulation of FOXO3a stability. The IBT results showed that only MG132 was able to enhance the stability of FOXO3a in the control group (Figure 5L). Then, ubiquitination-related immunoprecipitation specified that AIM2 decreased the ubiquitination level of FOXO3a in RCC cells (Figure 5M).

### FOXO3a directly transcriptionally regulated the expression of ACSL4 in RCC

To determine the relationship between FOXO3a and ACSL4, we predicted three binding sites for FOXO3a within 2000-bp upstream of ACSL4 transcription start site (Figure 5N). ChIP assays were performed in RCC cells to validate the exact sites. Compared with E3, E1 and E2 appeared to be more considerably enriched by the antiFOXO3a antibody (Figures 5O-5P). Furthermore, we constructed five truncated and mutant plasmids to explore which site mediated the transcriptional effect of FOXO3a on ACSL4 (Figure 5Q). We found that knocking down FOXO3a did not affect the detected luciferin levels as long as E1 was mutated (Figure 5R). Dual luciferase reporter experiments indicated that E1 was the main site of FOXO3a regulation of ACSL4. Therefore, we could draw the conclusion that AIM2 promoted FOXO3a phosphorylation and proteasome degradation, thereby reducing its transcriptional effect on ACSL4.

### Ferroptosis inhibited by AIM2 promoted sunitinib sensitivity of RCC

**Figure 6:**
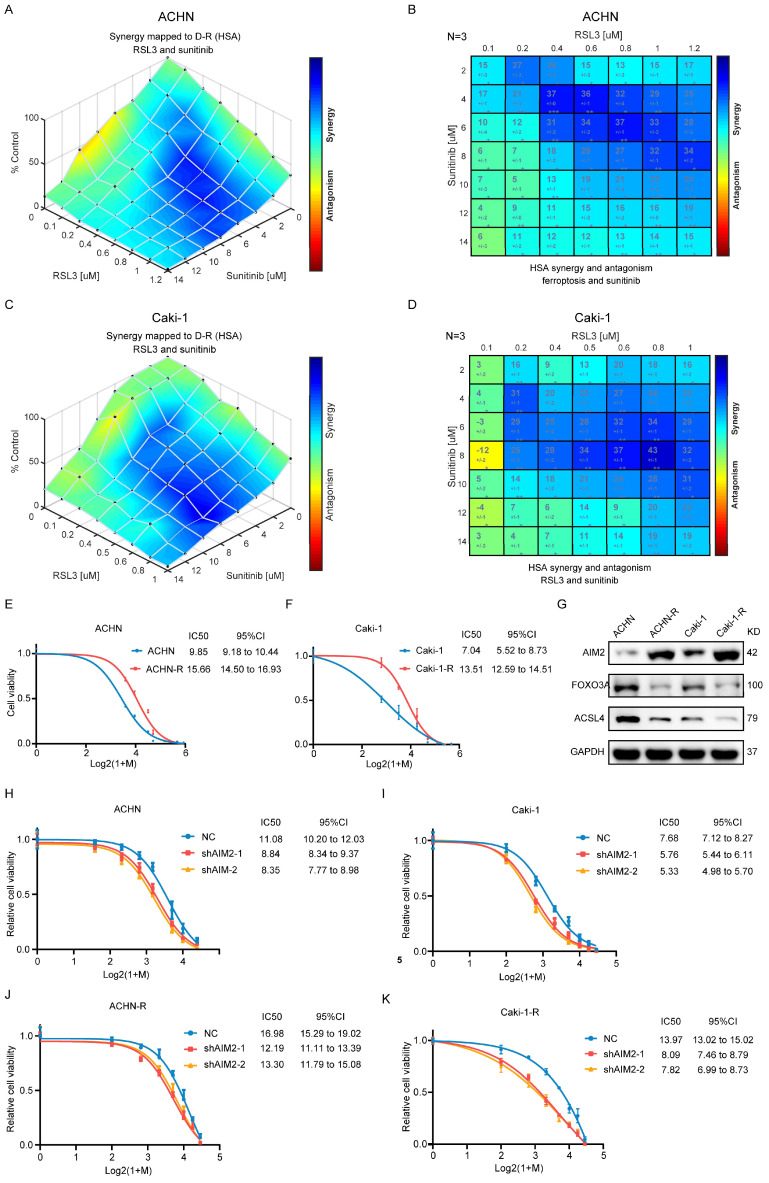
** Ferroptosis inhibited by AIM2 promoted the sunitinib sensitivity of RCC.** (A-D) Analysis and visualization of combination of RSL3 and sunitinib in RCC cells using combenefit software. (E-F) Sunitinib resistance curves for ACHN-R and Caki-1-R cells. (G) Protein levels of AIM2, FOXO3a and ACSL4 were detected in parental and sunitinib resistant RCC cells using IBT. (H-K) Sunitinib sensitivity curves for ACHN, Caki-1, ACHN-R and Caki-1-R cells with or without AIM2 knockdown.

The above-mentioned results suggested that AIM2 acted as an oncogene in RCC. In clinical practice, antiangiogenic therapy was a major treatment for difficult-to-operate RCC^24^. Although antiangiogenic therapy has shown considerable efficacy, most patients eventually developed resistance^25^. Sunitinib is the representative drug of clinical antiangiogenic therapy^26^. ACHN and Caki-1 cells showed a concentration-dependent increase in lipid peroxidation levels after being treated with sunitinib (Figures S6A and S6B), suggesting that sunitinib could promote ferroptosis in RCC. The results of GSEA based on TCGA-KIRC project indicated that AIM2 and FOXO3a were related with drug response and angiogenesis (Figures S6C-S6H). To explore the relationship between sunitinib resistance and ferroptosis regulated by AIM2-FOXO3a axis, a combination of different concentrations of RSL3 and sunitinib was employed in ACHN and Caki-1 cells. A drug combination analysis showed that RSL3 and sunitinib synergistically induced death in RCC cells (Figures 6A-6D and S6I-S6J). Then, sunitinib was used to construct sunitinib-resistant ACHN and Caki-1 cell lines (Figures 6E and 6F). Interestingly, the expression of AIM2 is elevated and that of FOXO3a and ACSL4 is reduced in sunitinib-resistant cells, which suggested suppressed ferroptosis in these cells (Figure 6G). Furthermore, as shown in figures 6H-6K, knockdown of AIM2 increased the sensitivity of sunitinib in sunitinib-resistant and normal RCC cell lines.

### AIM2 promoted RCC progression through FOXO3a *in vitro*

**Figure 7:**
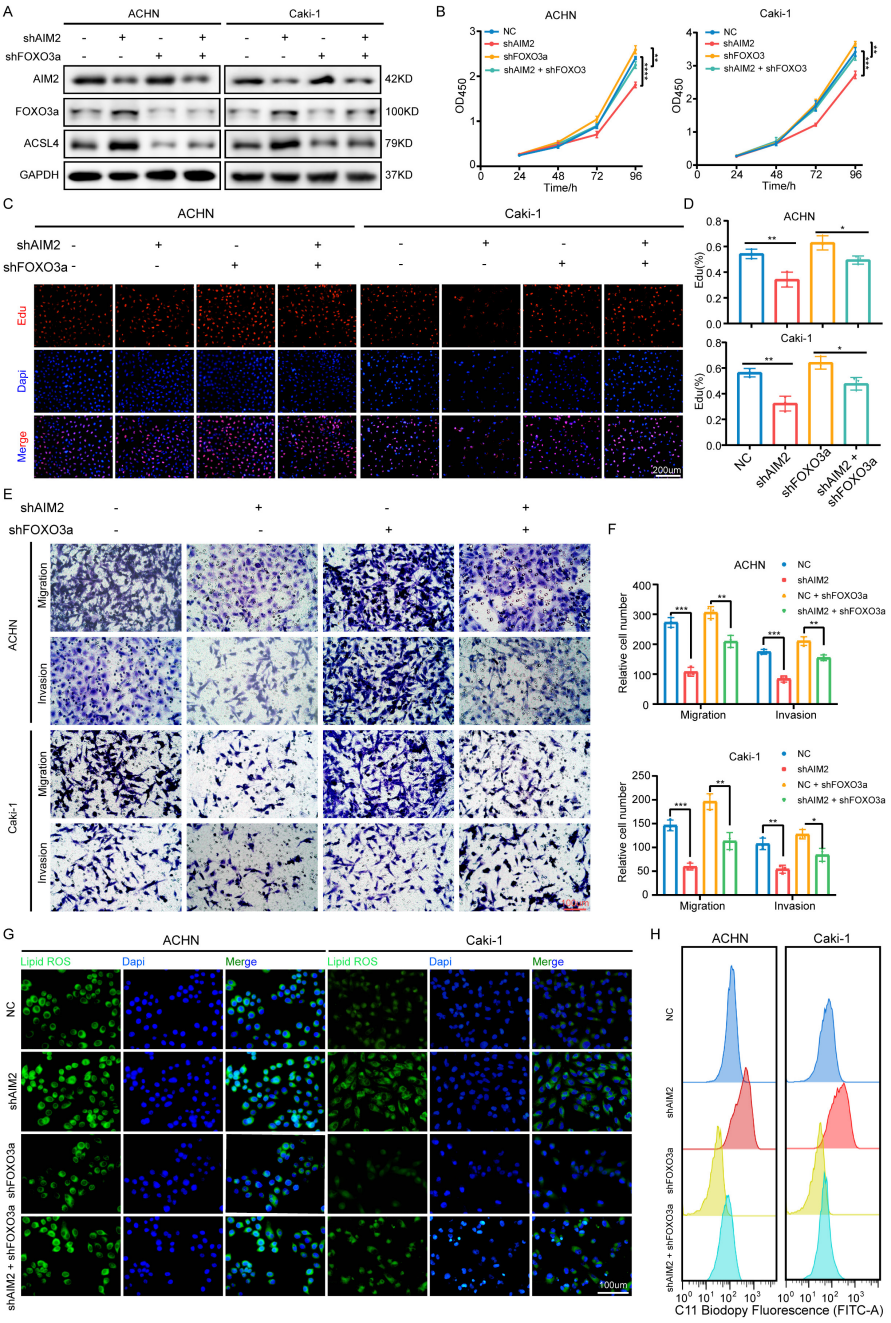
** AIM2 promoted RCC progression through FOXO3a *in vitro*.** (A) FOXO3a and ACSL4 protein levels in RCC cells with AIM2 and/or FOXO3a knockdown were shown by western blotting. (B) The growth curves of RCC cells with AIM2 and/or FOXO3a knockdown were determined by CCK8 assays (n = 4). (C-D) The proliferation ability of ACHN and Caki-1 cells with AIM2 and/or FOXO3a knockdown was measured using EdU assays (n = 3). (E-F) The metastasis ability of RCC cells with AIM2 and/or FOXO3a knockdown was determined by transwell assays (n = 3). (G) Using BODIPY C11 assays, lipid peroxidation levels in RCC cells with AIM2 and/or FOXO3a knockdown were determined. (H) Flow cytometry was used to determine lipid peroxidation level in ACHN and Caki-1 cells with AIM2 and/or FOXO3a knockdown. ****p < .0001, ***p < .001, **p < .01 and *p < .05.

To determine whether FOXO3a mediated the role of AIM2 in RCC, we knocked down FOXO3a using shFOXO3a-1 lentivirus in the AIM2 knockdown and negative control groups to perform recovery experiments. The IBT results showed that FOXO3a was the key intermediate molecule between AIM2 and ACSL4 (Figure 7A). The results of CCK-8 assays and EdU experiments showed that FOXO3a knockdown greatly alleviated the inhibition of RCC proliferation caused by AIM2 knockdown (Figures 7B-7D). Transwell experiments also revealed similar conclusions (Figures 7E and 7F). Furthermore, knockdown of FOXO3a effectively increased the level of lipid peroxidation induced by AIM2 knockdown in ACHN and Caki-1 cell lines (Figures 7G-7H and S7A-S7B).

### AIM2 promoted RCC progression through FOXO3a *in vivo*

**Figure 8:**
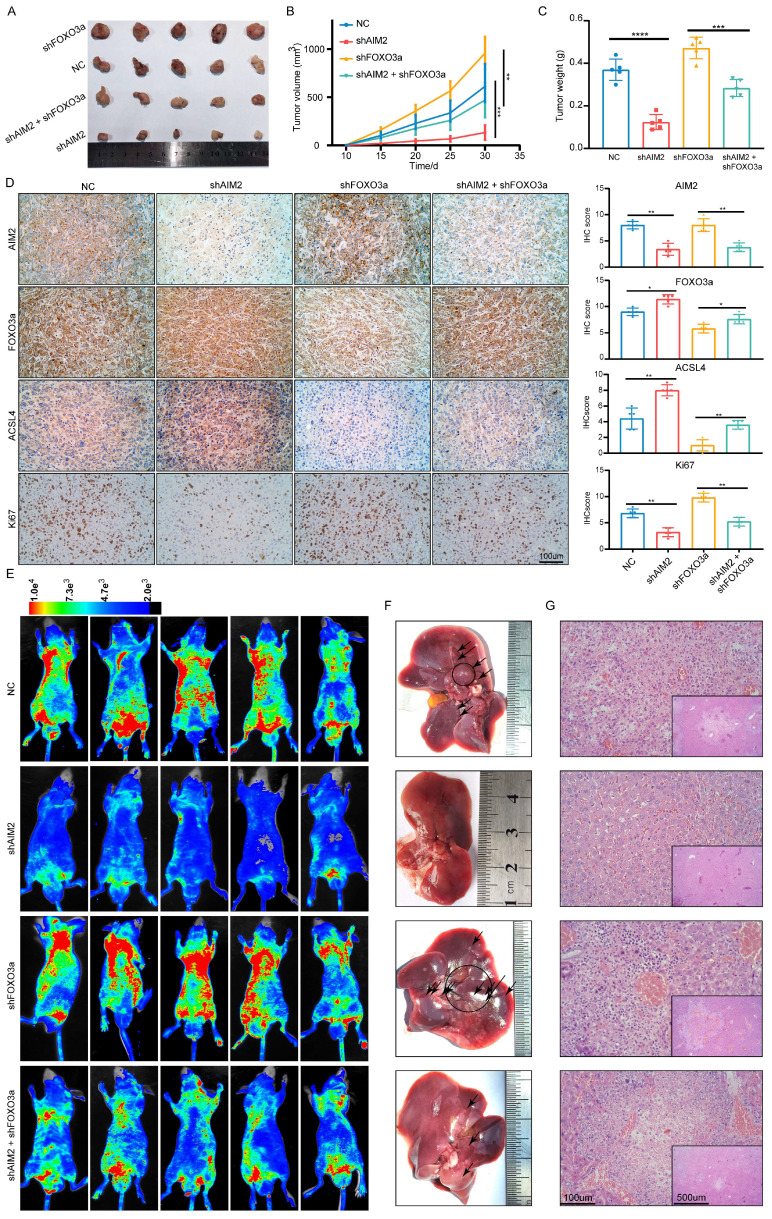
** AIM2 promoted RCC progression through FOXO3a *in vivo*.** (A) Pictures of xenografts formed from ACHN cells with AIM2 and/or FOXO3a knockdown (n = 5). (B) The growth curves of xenograft tumor in the four groups (n = 5). (B) Tumor weight of xenografts in the four groups (n = 5). (D) Immunohistochemical (IHC) staining and scores for AIM2, FOXO3a, ACSL4 and tumor malignancy (Ki67) in tumor xenografts. (E) Living fluorescence image in the metastasis mode from the four groups. (F-G) The picture and living fluorescence image of liver in the four groups. ****p < .0001, ***p < .001, **p < .01 and *p < .05.

To further prove that FOXO3a is an important gene for the promotion of RCC by AIM2 *in vivo*, Caki-1 cells with or without AIM2 knockdown were infected with shFOXO3a-1 lentivirus. We constructed xenograft tumor models using the above cell lines. Consistent with *in vitro* experiments, FOXO3a knockdown was shown to reverse the proliferation inhibition caused by AIM2 knockdown (Figures 8A-8C). The tumor malignant index Ki67 also confirmed this finding (Figure 8D). Subsequently, the immunohistochemistry assays of xenograft tumors showed that FOXO3a knockdown reversed the upregulation of ACSL4 caused by AIM2 inhibition (Figure 8D). Additionally, elevated lipid oxidation levels caused by AIM2 knockdown in xenograft tumors were markedly suppressed by FOXO3a inhibition (Figure S8A). Then, the tail vein metastasis models were constructed with the above Caki-1 cells. The results of fluorescence imaging of living mice and HE staining of livers showed that FOXO3a inhibition reversed the metastasis inhibition effect caused by AIM2 knockdown (Figures 8E-8G). In general, FOXO3a was an important downstream gene by which AIM2 promotes the progression of RCC. Collectively, our work can draw the following conclusions: elevated AIM2 levels in RCC promotes FOXO3a phosphorylation and proteasome degradation, thereby reducing its transcriptional effect on ACSL4; and reduced ACSL4 levels suppress ferroptosis, thereby promoting RCC progression and sunitinib resistance.

## Discussion

RCC is a prevalent type of malignant tumor of the urinary system that poses a major hazard to public health. The study of RCC progression and drug resistance mechanisms is particularly important clinically. In this investigation, we discovered AIM2 as a novel RCC biomarker. Further investigations showed that AIM2 could promote the progression of RCC and increase its sensitivity to sunitinib. Our findings may suggest a new target for RCC therapy.

As a receptor protein, AIM2 exerts distinct functions in different tumors in an inflammasome-dependent or -independent manner^18^. AIM2 plays a tumor-promoting role in skin and lung cancers and a tumor suppressor role in and colorectal cancers^8^, ^27^-^30^. For the first time, our study demonstrates that AIM2 promotes RCC independent of the inflammasome. A new mechanism was found, which demonstrates that AIM2 can inhibit ferroptosis in RCC through FOXO3a-ACSL4 axis. This provides new insights into the role of AIM2 in tumor research.

For inoperable RCC, TKI and immune checkpoint therapies are the first-line treatment options^31^, ^32^. As a common drug used in TKI therapy, sunitinib has shown marked clinical efficacy and improved the prognosis of many patients with RCC^33^. However, most RCC patients will inevitably eventually develop resistance to sunitinib^34^, ^35^. Sunitinib resistance has become the great challenge and difficulty faced in the clinical treatment of RCC. In our study, AIM2 was found to promote sensitivity to sunitinib in both resistant and normal RCC cell lines. Therefore, therapy targeting AIM2 is expected to become a complementary treatment for RCC.

Metabolic processes in tumors are very vigorous, producing many reactive oxygen species in tumor cells^36^. If not removed in time, excess reactive oxygen species in cells will combine with phospholipids containing PUFAs to form peroxidized lipids, which disrupts the membrane stability and ultimately leads to ferroptosis^37^. RCC contains abundant membranous organelles, such as mitochondria and endoplasmic reticulum; thus, it is sensitive to ferroptosis inducers^9^, ^38^. Some previous studies have suggested ferroptosis as a new target for the treatment of RCC^39^. Moreover, ferroptosis is thought to be associated with chemoresistance in tumors^11^, ^40^, ^41^. In our study, we were surprised to discover that the ferroptosis inducer RSL3 produced a synergistic effect with sunitinib. This means that ferroptosis may be involved in the drug effects of sunitinib and targeting ferroptosis is a new way to treat sunitinib resistance. Our future studies will focus on the mechanism by which ferroptosis is involved in the pharmacological effects of sunitinib.

## Conclusions

In conclusion, AIM2 was found to inhibit ferroptosis and promotes RCC progression and sunitinib resistance; thus, AIM2 could serve as a prognostic biomarker for RCC progression and a therapeutic target for RCC treatment.

## Supplementary Material

Supplementary figures and tables.

## Abbreviations

RCC: renal cell carcinoma
ccRCC: clear cell renal cell carcinoma
pRCC: papillary renal cell carcinoma
PFA: paraformaldehyde
TCGA: the Cancer Genome Atlas
WGCNA: weighted gene co-expression network analysis
ROC: receiver operator characteristic
qRT-PCR: reverse transcription-polymerase chain reaction
IHC: immunohistochemistry
ATCC: American Type Culture Collection
DMEM: Dulbecco's Modified Eagle's Medium
FBS: fetal bovine serum
IBT: Immunoblotting test
PVDF: polyvinylidene difluoride
FDR: false discovery rate
GSEA: gene set enrichment analysis
OS: overall survival
DFS: disease-free survival
KIRC: kidney renal clear cell carcinoma
CCK-8: Cell Counting Kit-8
NES: normalized enrichment score
CNV: copy number variation
SD: Standard Deviation
