# Supplementary material for: AIM2 promotes renal cell carcinoma progression and sunitinib resistance through FOXO3a-ACSL4 axis-regulated ferroptosis
Source: Int J Biol Sci. 2023 Feb 13;19(4):1266–83. doi: 10.7150/ijbs.79853 (PMC10008700; doi:10.7150/ijbs.79853)
Supplement: Supplementary file 1 — Supplementary figures and tables. [file ijbsv19p1266s1.pdf]

Supplementary tables
Table S1

Clinical characteristics of patients with RCC.

Characteristic	N (%)
Age	
Mean ± SEM, years	53±13
Sex	
Male/female	72/48
Tumor size	
Mean ± SEM, cm	5.8±3.2
Location	
Right/left	63/57
T stage	
T1a	26 (21.67)
T1b	53 (44.16)
T2a	16 (13.33)
T2b	11 (9.17)
T3	5 (4.17)
T4	2 (1.67)
Unknown	7 (5.83)
N stage	
N0	106 (88.33)
N1	14 (11.67)
M stage	
M0	109 (90.83)
M1	11 (9.17)
Fuhrman grade	
1	31 (25.83)
2	54 (45.00)
3	18 (15.00)
4	9 (7.50)
Unknown	8 (6.67)

SEM, standard error of the mean

Table S2

Antibody	manufacturer	Cat No.	species	application
GAPDH	Proteintech	10494-1-AP	rabbit	WB
AIM2	Abcam	not found	rabbit	WB
AIM2	Proteintech	66902-1-Ig	mouse	WB
FOXO3a	Abclonal	A0102	rabbit	WB，IP
FOXO3a	Cell signaling technology	D19A7	rabbit	IF，ChiP
P-FOXO3a	Abclonal	AP0684	rabbit	WB
ACSL4	Abclonal	A6828	rabbit	WB
ACSL4	Proteintech	66617-1-Ig	mouse	IF
ARTN	Cell signaling technology	5537	rabbit	WB
EP300	Cell signaling technology	86377	rabbit	WB
caspase-1	Cell signaling technology	24232	rabbit	WB
cleaved caspase-1	Cell signaling technology	D57A2	rabbit	WB

Gene	Forward	Reverse
GAPDH	CGTGGAAGGACTCATGACCA	GCCATCACGCCACAGTTTC
AIM2	TGAAACCCCGAAGATCAACAC	CCCAGTACTTCCATTTTCCCAG
FOXO3a	TCTACGAGTGGATGGTGCGTTG	CTCTTGCCAGTTCCCTCATTCTG
ACSL4	CATCCCTGGAGCAGATACTCT	TCACTTAGGATTTCCCTGGTCC
LOX	GCCGACCAAGATATTCCTGGG	GCAGGTCATAGTGGCTAAACTC
HMOX1	AAGACTGCGTTCCTGCTCAAC	AAAGCCCTACAGCAACTGTCG
FTH1	CCCCCATTTGTGTGACTTCAT	GCCCGAGGCTTAGCTTTCATT
KEAP1	CTGGAGGATCATACCAAGCAGG	GGATACCCTCAATGGACACCAC
NQO1	GAAGAGCACTGATCGTACTGGC	GGATACTGAAAGTTCGCAGGG
SLC7A11	TCTCCAAAGGAGGTTACCTGC	AGACTCCCCTCAGTAAAGTGAC
GPX4	GAGGCAAGACCGAAGTAAACTAC	CCGAACTGGTTACACGGGAA

Table S3

gene subset 1	
gene	Cor.geneModule Membership
IL2RB	0.824094658
LAG3	0.858403248
ICOS	0.825816164
TIGIT	0.878569431
SIRPG	0.90559738
PYHIN1	0.817853114
GZMA	0.846051683
GZMK	0.832832528
CD8A	0.834650354
BATF	0.908437486
IFNG	0.806012361
FASLG	0.831151998
AIM2	0.843204037
C1QB	0.818640358
CD3D	0.894961823
FCGR1B	0.860758506
FCGR1A	0.84982992
JAKMIP1	0.848640593
CD7	0.822414964
CXCR3	0.885489814
SIT1	0.887432573
PARVG	0.840484846
CST7	0.82692035
FCGR1C	0.835397292
CD2	0.890125664

gene subset2

gene	Cor.geneTraitSignificance				
	OS	neoplasm_histologic_grade	pathologic_M	pathologic_T	pathologic_stage
HAMP	0.25729	0.293930826	0.218957457	0.25544027	0.272208478
AURKB	0.31028	0.288244242	0.280944385	0.31099629	0.327575808
CXCL13	0.20954	0.329361907	0.230980623	0.31320772	0.312135011
CDC25C	0.24716	0.304616182	0.248246748	0.3133425	0.318796749
TYMP	0.21079	0.244928756	0.240086348	0.23823927	0.268143678
ALDH6A1	-0.31648	-0.257234173	-0.21305493	-0.3137571	-0.339628677
BATF	0.23171	0.306060889	0.21843659	0.27932996	0.288951067
MYBL2	0.27765	0.29372595	0.271087427	0.32679795	0.325295468
MUC12	0.21779	0.226649244	0.200665619	0.22018727	0.232077682
AIM2	0.22617	0.338613243	0.231750035	0.29471407	0.288907659
C7orf41	-0.2311	-0.277986664	-0.22142551	-0.28194813	-0.303769714
IL20RB	0.30309	0.234561123	0.293457024	0.30795257	0.350878616

TROAP	0.3182	0.254923637	0.271396615	0.32897484	0.331335301
FCGR1B	0.23562	0.321483877	0.207662079	0.27741593	0.27930862
UHRF1	0.2587	0.264644577	0.237915568	0.28106124	0.282710693
MSC	0.22835	0.285275246	0.231677289	0.23652349	0.26716553

Table S4

Correlation between AIM2 mRNA expression and clinicopathological parameters of RCC patients.

**Parameter**		**AIM2 mRNA expression**		
	**Number**	**Low (n = 261)**	**High (n = 261)**	**P-value**
**Age (years)**				
< 60	242	128	114	0.2538
≥ 60	280	133	147	
**Gender**				
Female	183	101	82	0.0986
Male	339	160	179	
**T stage**				
T1 or T2	336	189	147	0.0002
T3 or T4	186	72	114	
**N stage**				
N0 or Nx	507	258	249	0.0326
N1	15	3	12	
**M stage**				
M0 or Mx	445	237	208	0.0005
M1	77	24	53	
**G grade**				
G1 or G2 or Gx	235	150	85	<0.0001
G3 or G4	277	111	166	
**TNM stage**				
I + II	318	182	136	<0.0001
III + IV	204	79	125	

Table S5

Univariate and multivariate analyses of AIM2 mRNA level and patient overall survival (OS)

Variable	Univariate analysis			Multivariate analysis[c]		
	HR[a]	95% CI[b]	P	HR	95% CI	*P*
**Overall survival (n = 522)**						
**AIM2**						
Low (n = 261)	1.814	1.327-2.479	<0.001	1.434	1.04-1.978	0.028
High (n = 261)						
**Age**						
＜60 (n = 235)	1.736	1.265-2.382	0.001	1.532	1.112-2.111	0.009
⩾60 (n = 287)						
**Gender**						
Female (n = 183)	1.071	0.783-1.464	0.669			
Male (n = 339)						
**T stage**						
T1 or T2 (n = 336)	3.18	2.337-4.326	<0.001			
T3 or T4 (n = 186)						
**N stage**						
N0 or NX (n = 507)	3.96	2.143-7.315	<0.001	2.089	1.11-3.929	0.022
N1 (n = 15)						
**M stage**						
M0 or MX (n = 445)	4.378	3.199-5.992	<0.001	2.27	1.567-3.289	<0.001
M1 (n = 77)						
**G grade**						
Gx or G1 or G2 (n = 245)	2.681	1.901-3.782	<0.001	1.657	1.149-2.390	0.007
G3 or G4 (n = 277)						

a, Hazard ratio, estimated from Cox proportional hazard regression model; b, Confidence interval of the estimated HR; c, Multivariate models were adjusted for T, N, M classification, age, and gender.

Supplementary figures and figure legends

A

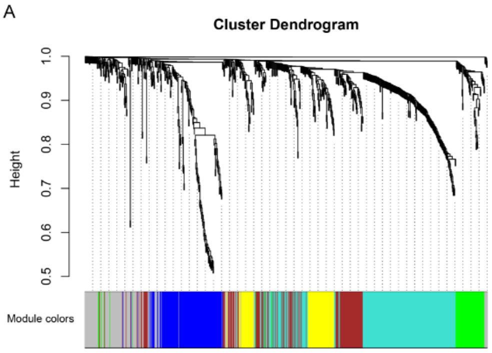

B

gene symble	Cor.geneModule Membership	Cor.geneTraitSignificance				
		OS	neoplasm_histologic_grade	pathologic_M	pathologic_T	pathologic_stage
AIM2	0.84	0.23	0.34	0.23	0.29	0.29
BATF	0.91	0.23	0.31	0.22	0.28	0.29
FCGR1B	0.86	0.24	0.32	0.21	0.28	0.28

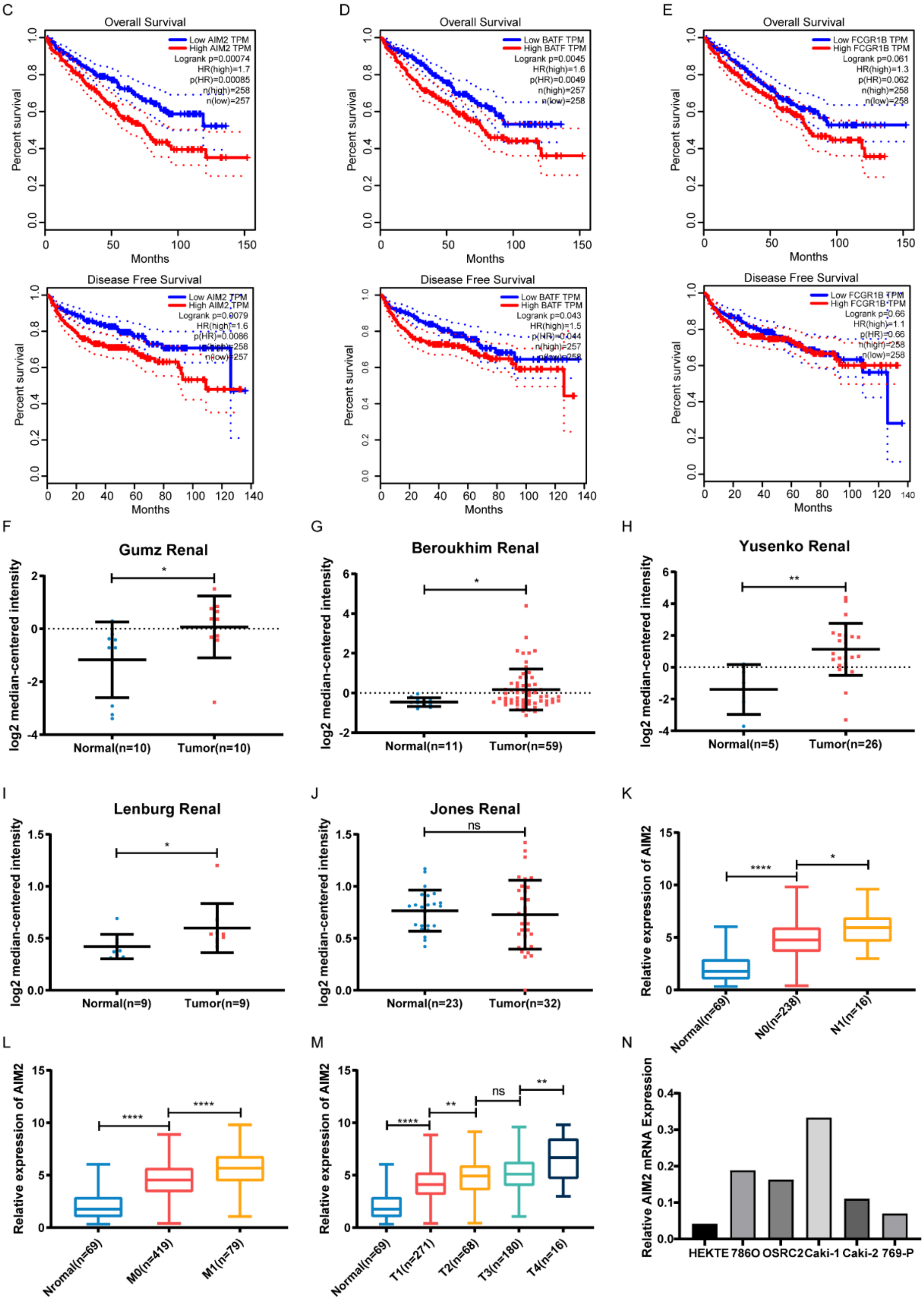

Figure S1 AIM2 was as a biomarker of RCC

(A) The six gene dendrogram and module colors of DEGs based on TOM. (B) The three genes from two gene sets. (C-E) The OS and DFS curves of AIM2, BATF and FCGR1B. All patients are classified according to the corresponding gene expression and then divided into two groups. OS and DFS in both groups of patients were visualized by Kaplan–Meier plot. OS: overall survival; DFS: disease free survival. (F-J) The overexpressed AIM2 in RCC from Gumz Renal, Beroukhim Renal, Yusenko Renal, Lenburg Renal and Jones Renal using Oncomine database. (K-M) Expression of AIM2 according to N, M and T stage in TCGA-KIRC project. (N) The expression of AIM2 according to CCLE database.

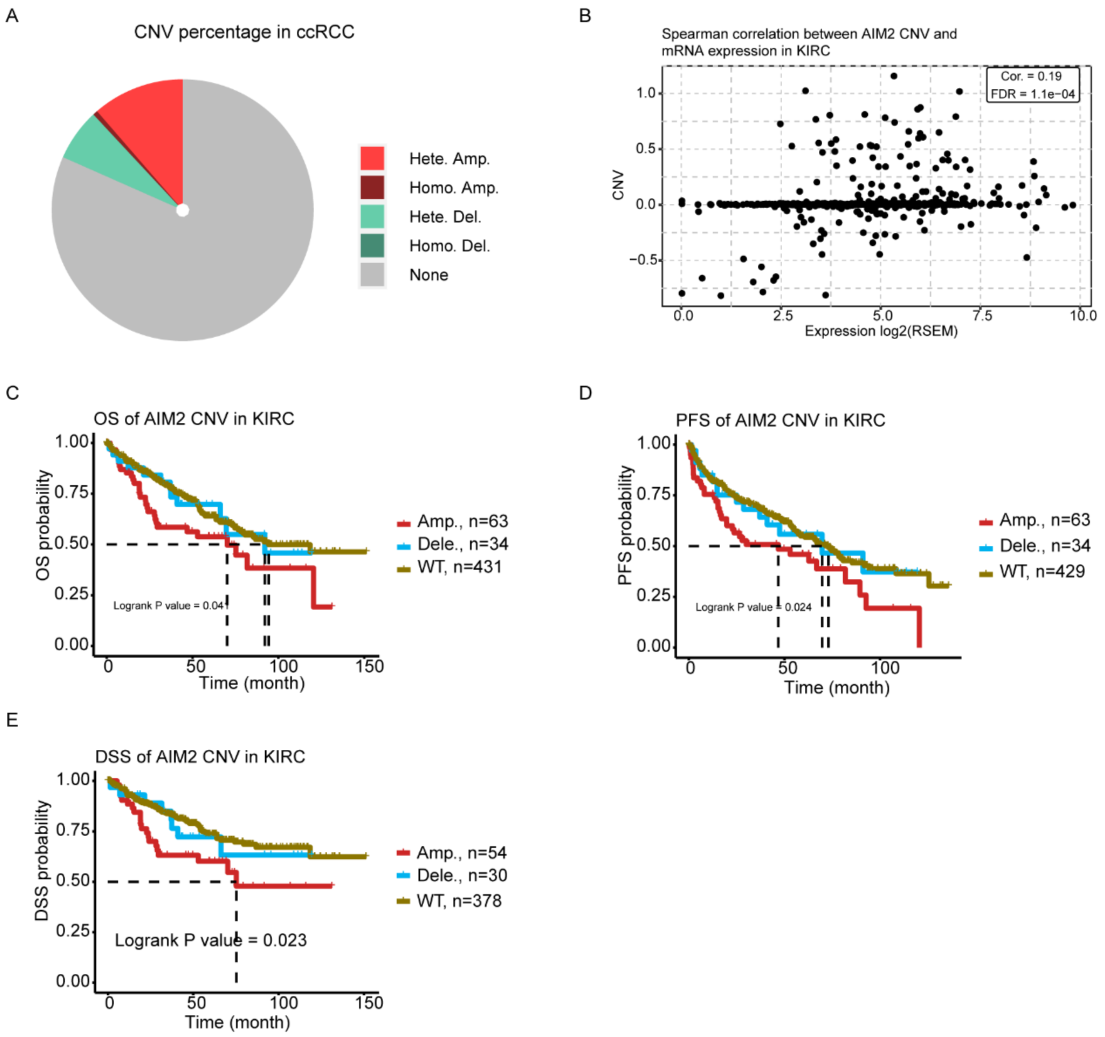

Figure S2 Overexpression of AIM2 was related to its amplified copy number variation.

(A) Pie chart showed CNV percentage in RCC. (B) Spearman correlation between AIM2 CNV and mRNA expression in KIRC. (C-E) The OS, PFS and DSS curves of AIM2 CNV were visualized by Kaplan–Meier plot. CNV: copy number variation.

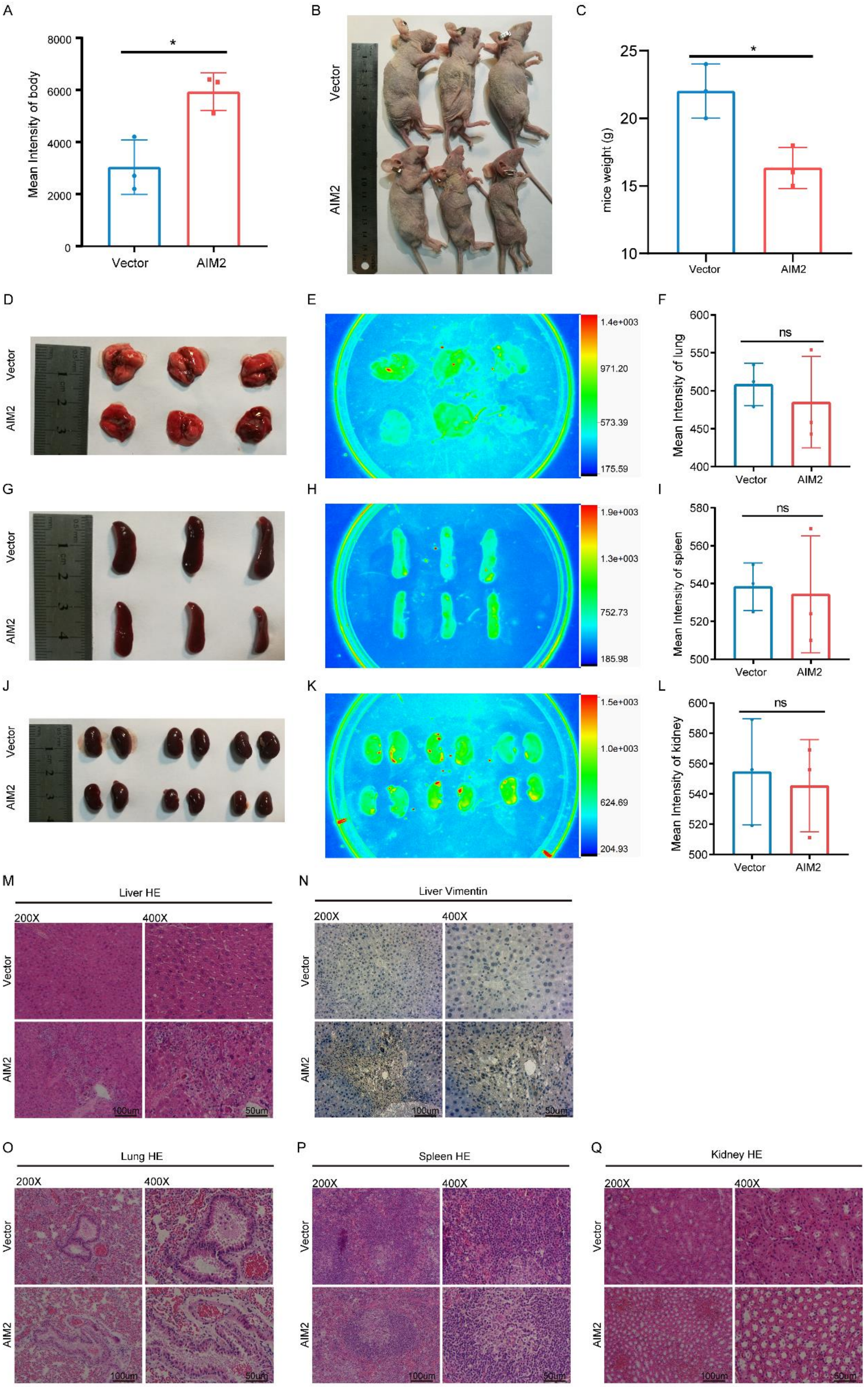

Figure S3 AIM2 promoted RCC progression in vivo

(A) Statistical chart of fluorescent intravital imaging in mice (n = 3). (B) Photos of mice in the metastasis model. (C) The weight of mice in the metastasis model. (D-L) Photos and tissues fluorescence imaging of lung, spleen, and kidney. (M, O, P and Q) HE staining of liver, lung, spleen, and kidney in vector and AIM2 overexpression groups. (N) The vimentin staining of liver in vector and AIM2 overexpression groups.

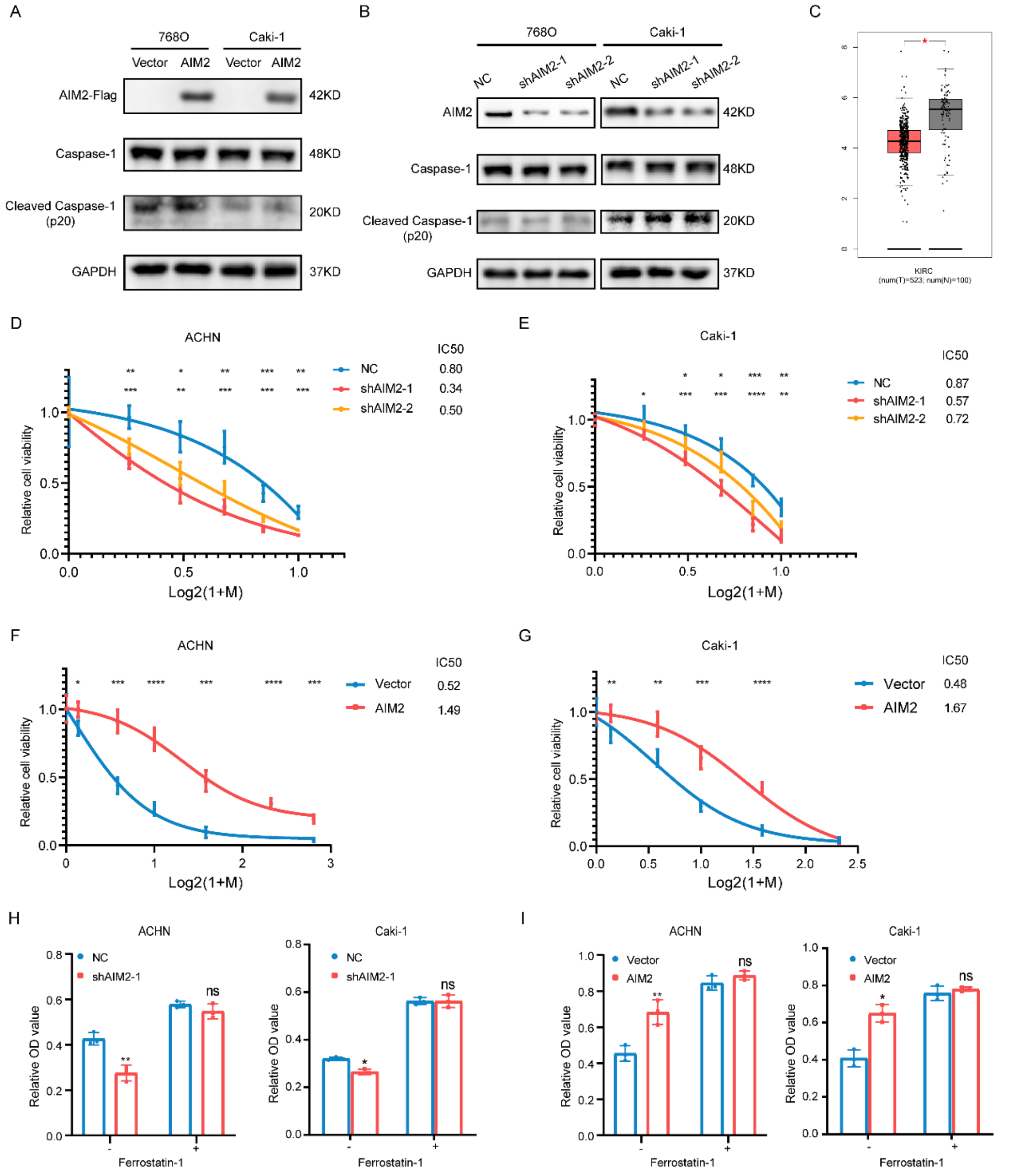

Figure S4 AIM2 Inhibited ferroptosis in RCC

(A-B) The caspase-1 and cleaved caspase-1 protein levels in ACHN and Caki-1 cells

with AIM2 overexpression or knockdown. (C) The mRNA expression of ACSL4 in TCGA-KIRC project. (D-G) The RSL3 sensitive curves of ACHN and Caki-1 cells with AIM2 knockdown and overexpression. (H-I) After treated with ferrostatin-1, the cell viability of ACHN and Caki-1 cells with AIM2 knockdown or overexpression.

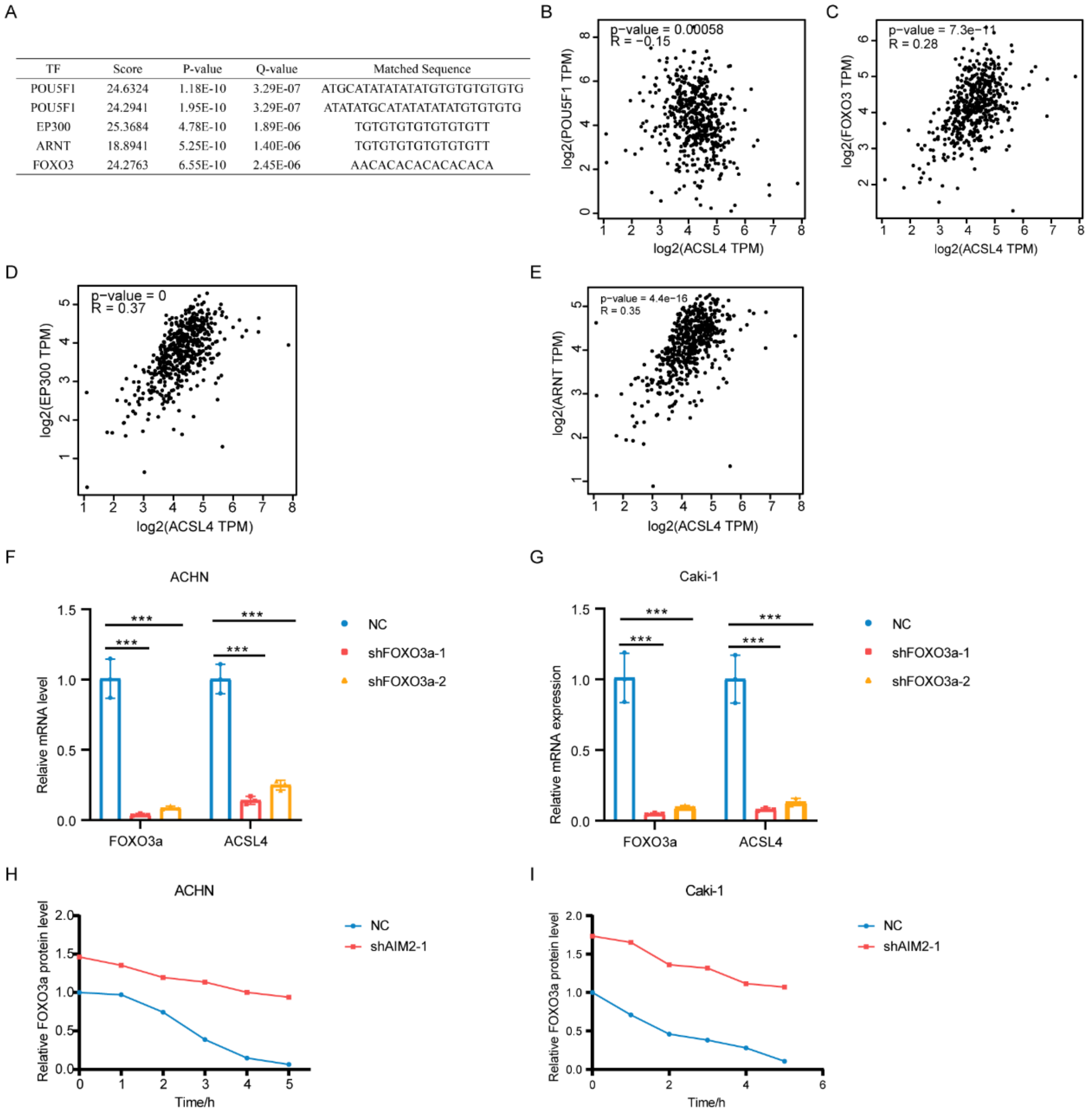

TF	Score	P-value	Q-value	Matched Sequence
POU5F1	24.6324	1.18E-10	3.29E-07	ATGCATATATATATGTGTGTGTGTG
POU5F1	24.2941	1.95E-10	3.29E-07	ATATATGCATATATATATGTGTGTG
EP300	25.3684	4.78E-10	1.89E-06	TGTGTGTGTGTGTGTT
ARNT	18.8941	5.25E-10	1.40E-06	TGTGTGTGTGTGTGTT
FOXO3	24.2763	6.55E-10	2.45E-06	AACACACACACACACA

Figure S5 AIM2 inhibited ACSL4 activity through FOXO3a

(A) The potential top five transcription factors using the animal TFDB database. (B) The expression of POU5F1 is negatively correlated to the expression of AIM2 using GEPIA database according to TCGA-KIRC project. (C-E) The expression of FOXO3a, EP300 and ARTN was positively correlated to expression of AIM2 in TCGA-KIRC

project. (F-G) The mRNA level of ACSL4 was detected by qPCR in ACHN and caki-1 cells. (H-I) The protein content of FOXO3a was quantified using Image Lab software.

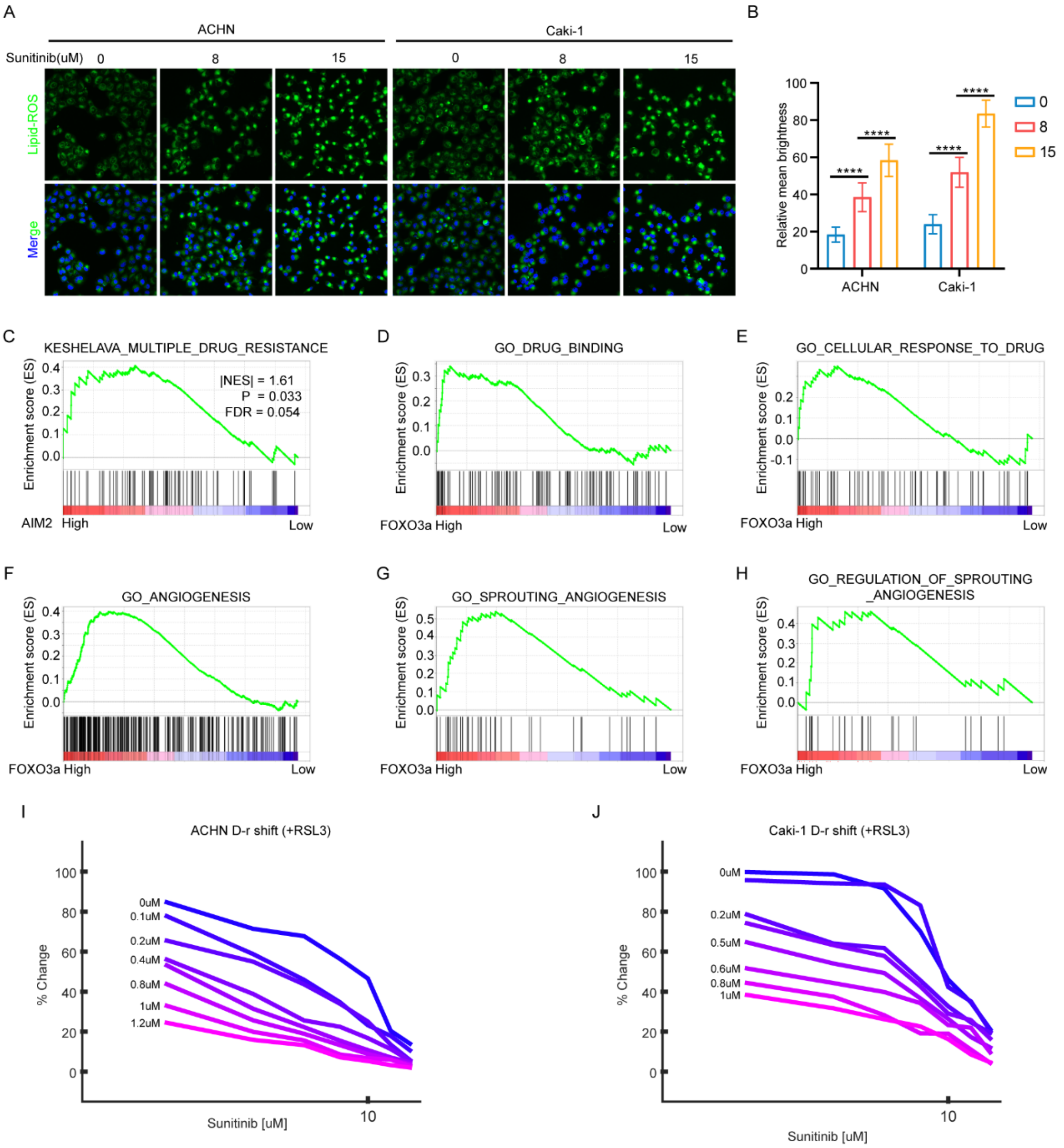

Figure S6 Ferroptosis inhibited by AIM2 promoted the sunitinib sensitivity of RCC

(A-B) After being treated with sunitinib, the lipid-ROS levels of ACHN and Caki-1 cells were visualized. Fluorescence brightness was counted using image J software, with at least 30 cells participating in the count. (C-H) The GSEA results according to the expression of AIM2 and FOXO3a in TCGA-KIRC project at the criteria of FDR less than 0.25, P less than 0.05, |NES| > 1. (I-J) The sunitinib sensitive curves of ACHN

and Caki-1 cells with different concentrations of RSL3. FDR: false discovery rate, NES: normalized enrichment score.

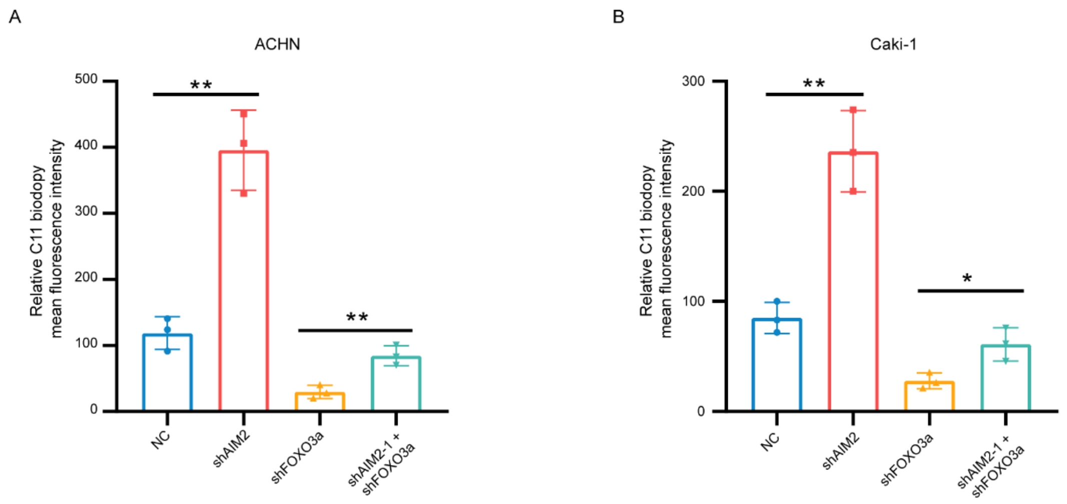

Figure S7 AIM2 promoted RCC progression through FOXO3a in vitro

(A-B) The relative C11 bodipy mean fluorescence intensity of ACHN and Caki-1 cells with AIM2 and/or FOXO3a knockdown.

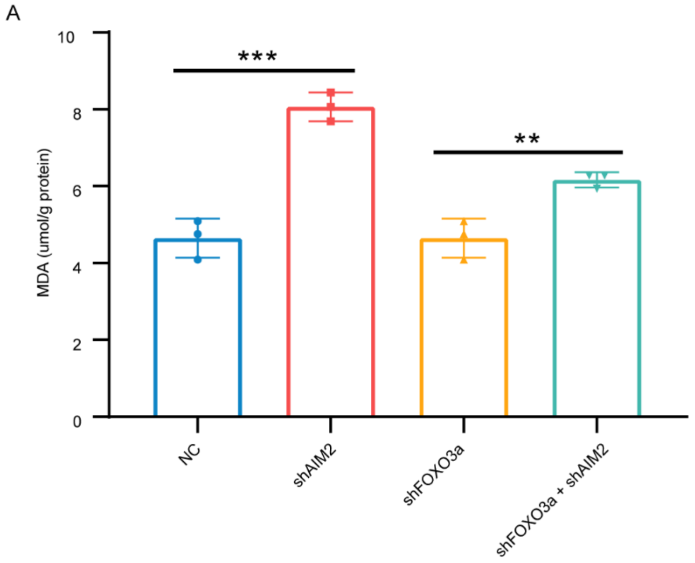

Figure S8 AIM2 promoted RCC progression through FOXO3a in vivo

(A) The relative MDA levels of xenograft tumors with AIM2 and/or FOXO3a knockdown.
